# Retinoic Acid-Related Orphan Receptor γ (RORγ): A Novel Participant in the Diurnal Regulation of Hepatic Gluconeogenesis and Insulin Sensitivity

**DOI:** 10.1371/journal.pgen.1004331

**Published:** 2014-05-15

**Authors:** Yukimasa Takeda, Hong Soon Kang, Johannes Freudenberg, Laura M. DeGraff, Raja Jothi, Anton M. Jetten

**Affiliations:** 1 Cell Biology Section, Division of Intramural Research, National Institute of Environmental Health Sciences, National Institutes of Health, Research Triangle Park, North Carolina, United States of America; 2 Systems Biology Group, Division of Intramural Research, National Institute of Environmental Health Sciences, National Institutes of Health, Research Triangle Park, North Carolina, United States of America; Charité - Universitätsmedizin Berlin, Germany

## Abstract

The hepatic circadian clock plays a key role in the daily regulation of glucose metabolism, but the precise molecular mechanisms that coordinate these two biological processes are not fully understood. In this study, we identify a novel connection between the regulation of RORγ by the clock machinery and the diurnal regulation of glucose metabolic networks. We demonstrate that particularly at daytime, mice deficient in RORγ exhibit improved insulin sensitivity and glucose tolerance due to reduced hepatic gluconeogenesis. This is associated with a reduced peak expression of several glucose metabolic genes critical in the control of gluconeogenesis and glycolysis. Genome-wide cistromic profiling, promoter and mutation analysis support the concept that RORγ regulates the transcription of several glucose metabolic genes directly by binding ROREs in their promoter regulatory region. Similar observations were made in liver-specific RORγ-deficient mice suggesting that the changes in glucose homeostasis were directly related to the loss of hepatic RORγ expression. Altogether, our study shows that RORγ regulates several glucose metabolic genes downstream of the hepatic clock and identifies a novel metabolic function for RORγ in the diurnal regulation of hepatic gluconeogenesis and insulin sensitivity. The inhibition of the activation of several metabolic gene promoters by an RORγ antagonist suggests that antagonists may provide a novel strategy in the management of metabolic diseases, including type 2 diabetes.

## Author Summary

The circadian clock plays a critical role in the regulation of many physiological processes, including metabolism and energy homeostasis. The retinoic acid-related orphan receptor γ (RORγ) functions as a ligand-dependent transcription factor that regulates transcription by binding as a monomer to ROR-responsive elements. In liver, RORγ exhibits a robust circadian pattern of expression that is under direct control of the hepatic circadian clock. However, the connection between the circadian regulation of RORγ and its control of downstream metabolic processes is not well understood. In this study, by using ubiquitous and liver-specific *RORγ*-deficient mice as models, we demonstrate that hepatic RORγ modulates daily insulin sensitivity and glucose tolerance by regulating hepatic gluconeogenesis. Genome-wide cistromic profiling, gene expression, and promoter analysis revealed that RORγ is targeting and regulating a number of novel metabolic genes critical in the control of glycolysis and gluconeogenesis pathways. We provide evidence for a model in which RORγ regulates the circadian expression of glucose metabolic genes in the liver downstream of the hepatic circadian clock, thereby enhancing gluconeogenesis and decreasing insulin sensitivity and glucose tolerance. This study suggests that attenuating RORγ activity by antagonists might be beneficial for the management of glucose metabolic diseases including type 2 diabetes.

## Introduction

RORγ constitutes with RORα and RORβ, the retinoic acid-related orphan receptor (ROR; NR1F1–3) subfamily of the nuclear receptors, which regulate transcription by binding as monomers to ROR-responsive elements (ROREs) in the regulatory region of target genes [1], [2]. Through alternative promoter usage, the *RORγ* gene generates 2 isoforms, *RORγ1* and *RORγ2* (*RORγt*), that regulate different physiological functions. *RORγt* is restricted to several distinct immune cells and is essential for thymopoiesis, lymph node development, and Th17 cell differentiation [1], [3]–[5]. RORγ antagonists inhibit Th17 cell differentiation and may provide a novel therapeutic strategy in the management of several autoimmune diseases [4], [6].

In contrast to RORγt, relatively little is known about the physiological functions of RORγ1. The expression of RORγ1 is highly restricted to tissues that have major functions in metabolism and energy homeostasis, including liver and adipose tissue, and in contrast to RORα and RORβ, RORγ is not expressed in the central nervous system, including the hypothalamus and suprachiasmatic nucleus [1], [6]–[13]. In several peripheral tissues *RORγ1* exhibits a robust rhythmic pattern of expression with a peak at zeitgeber time (ZT) 16–20 that is directly regulated by the clock proteins, brain and muscle ARNT-like (Bmal1) and circadian locomotor output cycles kaput (Clock), and the Rev-Erb nuclear receptors [1], [8]–[12], [14], [15]. Although RORγ is recruited to ROREs in the regulatory regions of several clock genes, including *Bmal1*, *Clock*, *Rev-Erbα*, and cryptochrome 1 (*Cry1*); the loss of RORγ has little influence on the expression of *Bmal1* and *Clock*, and only modestly reduces the expression of *Rev-Erbα* and *Cry1* [10], [12]; The robust oscillatory regulation of RORγ1 expression by the clock machinery raised the possibility that RORγ might regulate the expression of certain target genes in a ZT-dependent manner. Because the clock machinery plays a critical role in the circadian regulation of many metabolic pathways, including glucose metabolism [13], [16]–[19], RORγ may function as an intermediary between the clock machinery and the regulation of metabolic genes. Since recent studies indicated an association between the level of RORγ expression and obesity-associated insulin resistance in mice and humans [20], [21], these observations led us to propose that RORγ1 might be an important participant in the diurnal regulation of glucose metabolic pathways [10], [16], [18], [22].

To study this hypothesis further, we examined the effect of the loss of RORγ on the diurnal regulation of glucose metabolism in ubiquitous and the hepatocyte-specific RORγ knockout mice. This analysis showed that loss of RORγ enhances glucose tolerance and insulin sensitivity particularly during early daytime (ZT4–6) and reduces the peak expression of several glucose metabolic genes. RORγ cistrome and promoter analysis indicated that several of these metabolic genes were regulated directly by RORγ and involved ZT-dependent recruitment of RORγ to ROREs in their regulatory region. Together, our observations are consistent with the concept that RORγ directly regulates the diurnal expression of a number of glucose metabolic genes in the liver downstream of the hepatic clock machinery, thereby enhancing gluconeogenesis and decreasing insulin sensitivity and glucose tolerance. The inhibition of the activation of several glucose metabolic gene promoters by an RORγ antagonist suggests that such antagonists might provide a novel therapeutic strategy in the management of insulin resistance and type 2 diabetes.

## Results

### Loss of RORγ improves insulin sensitivity and glucose tolerance in a ZT-dependent manner

**Figure 1:**
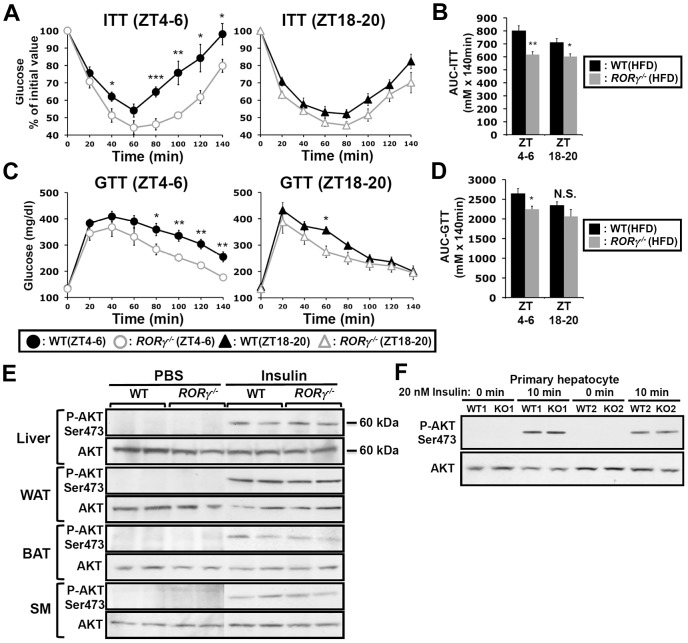
Loss of RORγ improves insulin and glucose tolerance in a ZT-dependent manner. ITT (**A**) and GTT (**C**) were examined at ZT4–6 and ZT18–20 in WT and *RORγ^−/−^* mice fed a HFD for 6 weeks (n = 7–12). Data represent mean ±SEM, * P<0.05, ** P<0.01, *** P<0.001 by ANOVA. (**B, D**) Comparison of AUC for ITT and GTT by one way ANOVA. AUC was also calculated by 2-way ANOVA; for ITT: Time period P = 0.080 and Genotype P = 0.0002; for GTT: Time period P = 0.073 and Genotype P = 0.013 (not shown). (**E**) Loss of RORγ did not affect Akt activation. Total and phosphorylated of Akt were examined by Western blot analysis in liver, BAT, WAT, and skeletal muscle (SM) isolated from WT(HFD) and *RORγ^−/−^*(HFD) mice 30 min after intraperitoneal injection of either 0.75 U/kg insulin or PBS. (**F**) Representative Western blot analysis (n = 2) of total and phosphorylated Akt in primary mouse hepatocytes isolated from WT and *RORγ^−/−^* mice. Cells were treated with 20 nM insulin or PBS for 10 min before proteins were isolated.

Glucose tolerance and insulin sensitivity, as RORγ1 expression, have been reported to be under endogenous circadian control [23], [24]. Recently, we proposed that RORγ1 might be an important participant in the diurnal regulation of several glucose metabolic pathways downstream of the circadian clock [10], [22]. To study the potential role of RORγ in glucose homeostasis, we examined the effect of the loss of RORγ on insulin sensitivity, glucose tolerance and the rhythmic expression pattern of glucose metabolic genes in ubiquitous and hepatocyte-specific RORγ knockout mice. Our data revealed that the loss of RORγ expression had a significant effect on insulin tolerance (ITT) and glucose tolerance (GTT) in mice fed with a high-fat diet (HFD). Comparison of the insulin responsiveness at two different time periods, ZT4–6 (daytime) and ZT18–20 (nighttime) showed that in wild type mice fed a HFD (WT(HFD)) insulin was more effective in controlling glucose levels at ZT18–20 than at ZT4–6 indicating that insulin sensitivity was ZT dependent [23], [24] (Figure 1A). Interestingly, this ZT-dependent difference in insulin responsiveness was greatly diminished in *RORγ^−/−^*(HFD) mice. ITT analysis showed that at ZT4–6 blood glucose levels remained significantly lower in *RORγ^−/−^*(HFD) mice after insulin injection than in WT(HFD) mice particularly after reaching a trough at 60 min (Figure 1A and Table S1). ITT performed at CT4–6 under constant darkness similarly showed improved insulin sensitivity in *RORγ^−/−^*(HFD) mice (Figure S1A), suggesting that RORγ significantly affects insulin sensitivity also under a Zeitgeber-free condition. At ZT18–20 the difference in ITT response between WT(HFD) and *RORγ^−/−^*(HFD) mice was significantly smaller than at ZT4–6. Consistent with the improved insulin sensitivity, GTT analysis showed that *RORγ^−/−^*(HFD) mice were more glucose tolerant than WT(HFD) particularly at ZT4–6 (Figure 1C). Although the difference was smaller than in mice fed with a HFD, *RORγ^−/−^*(ND) mice fed with a normal diet (ND) were also significantly more insulin sensitive and glucose tolerant at ZT4–6 than WT(ND) mice (Figure S1C and S1D). Because of the larger difference in mice fed a HFD, we focused much of our further analysis particularly on these mice. Altogether our observations indicate that the loss of RORγ enhanced glucose tolerance and insulin sensitivity particularly at ZT4–6 and CT4–6. Analysis of the areas under the curves (AUC) for ITT and GTT was consistent with this conclusion (Figure 1B and 1D).

To obtain further insights into the improved insulin sensitivity in *RORγ^−/−^* mice, we compared the level of insulin-induced activation of Akt phosphorylation (P-Akt), one of the most sensitive phosphorylation targets in the insulin signaling pathway, in liver and several other metabolic tissues (Figure 1E). No significant difference in P-Akt was observed at ZT4–6 in liver, brown and white adipose tissue (BAT, WAT), skeletal muscle between WT(HFD) and *RORγ^−/−^*(HFD) mice after insulin stimulation. Moreover, no significant difference in P-Akt was observed between insulin-treated WT and *RORγ^−/−^* primary hepatocytes (Figure 1F). These results suggest that loss of RORγ does not alter insulin-dependent phosphorylation of Akt in several metabolic tissues.

### RORγ participates in the diurnal regulation of hepatic gluconeogenesis

**Figure 2:**
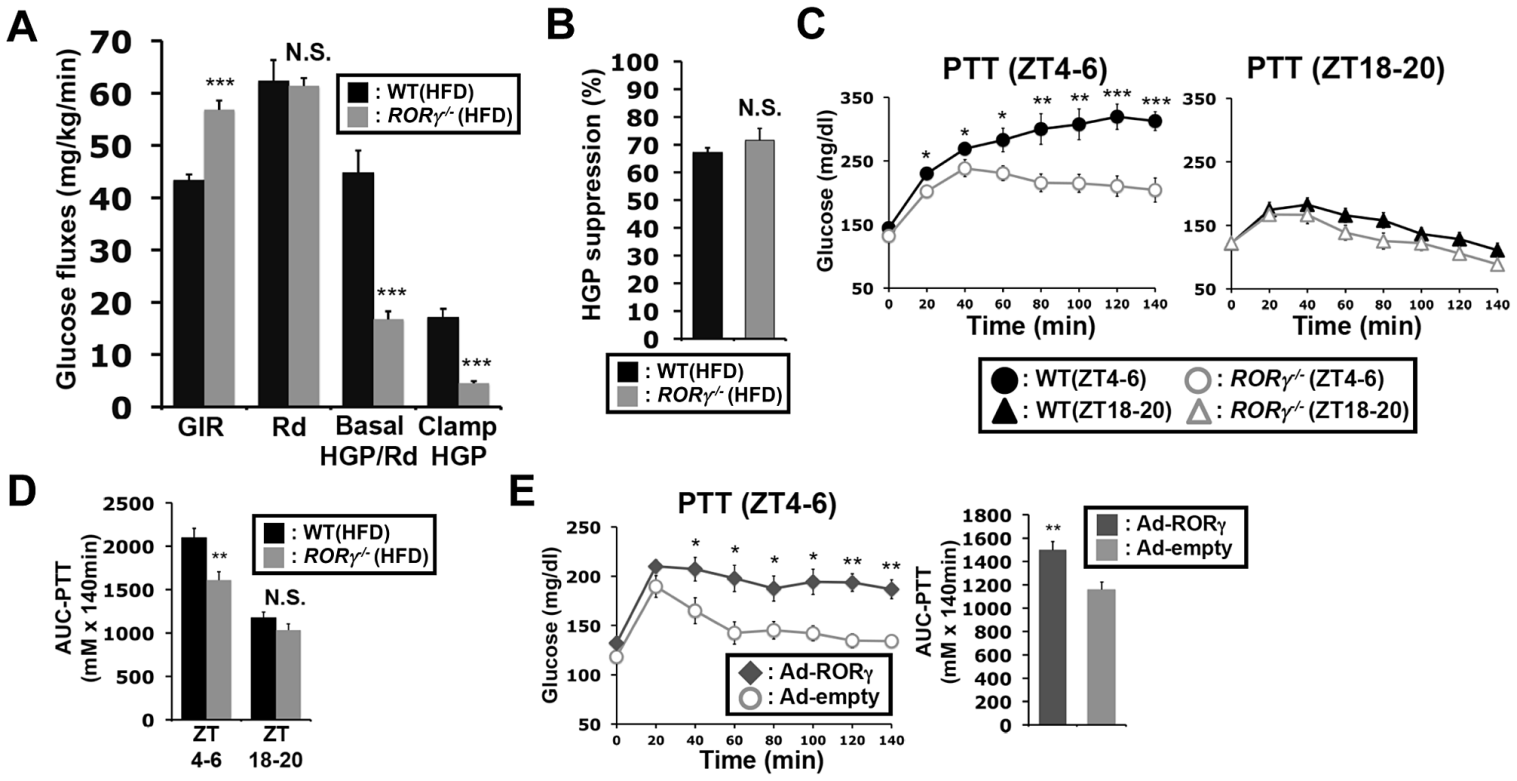
Loss of RORγ leads to reduced hepatic gluconeogenesis at daytime. (**A**) The hyperinsulinemic-euglycemic clamp test was performed during daytime (ZT2–9) in WT(HFD) and *RORγ^−/−^*(HFD) mice and the glucose infusion rate (GIR), whole-body glucose disappearance (Rd), basal endogenous hepatic glucose production (Basal HGP), and endogenous hepatic glucose production during the clamp (Clamp HGP) were determined. (**B**) Suppression rate of hepatic glucose production by insulin in WT(HFD) and *RORγ^−/−^*(HFD) mice. (**C**) PTT was examined at ZT4–6 and ZT18–20 in WT(HFD) and *RORγ^−/−^*(HFD) mice (n = 8) as indicated. (**D**) Comparison of AUC for PTT by one way ANOVA. AUC for PTT was also evaluated by 2-way ANOVA: Time period, P = 0.0001; Genotype, P = 0.0009 (not shown). (**E**) PTT was examined at ZT4–6 in *RORγ^−/−^*(HFD) mice injected with either empty or RORγ-containing adenovirus injection (n = 6). Data represent mean ±SEM, * P<0.05, ** P<0.01, *** P<0.001 by ANOVA.

Next, we examined insulin sensitivity and glucose fluxes at daytime by the hyperinsulinemic-euglycemic clamp test. Consistent with the results of ITT, the glucose infusion rate (GIR) required to maintain blood glucose level under constant insulin infusion was significantly higher in *RORγ^−/−^*(HFD) mice than in WT(HFD) mice at daytime (ZT2–9), while their glucose absorption rate estimated by whole-body glucose disappearance (Rd) was almost equal during the clamp (Figure 2A, S2A, S2B). Importantly, basal hepatic glucose production (HGP) and clamp HGP were significantly lowered in *RORγ^−/−^* mice. Insulin equally suppressed the HGP about 70% in both WT and *RORγ^−/−^* mice (Figure 2B), indicating that the insulin responsiveness was not changed in *RORγ^−/−^* mice, consistent with the observation in Figures 1E and 1F. Glucose turnover estimated from the steady-state infusion of ^3^H-glucose (Basal HGP and Rd) [25] was lower in *RORγ^−/−^* mice, indicating that the glucose absorption rate might also be reduced. These results suggest that the increased GIR required to maintain blood glucose level in *RORγ^−/−^* mice was due to reduced hepatic glucose production and not due to improved insulin responsiveness.

The clamp test suggested that the output of hepatic glucose produced by gluconeogenesis and glycogenolysis was reduced in *RORγ^−/−^* mice. Because hepatic gluconeogenesis is under close control of the circadian clock [18], [23], [26], we analyzed gluconeogenesis efficiency at 2 different ZTs in WT and *RORγ^−/−^* mice fed with either a ND or HFD. The pyruvate tolerance test (PTT) indicated that gluconeogenesis was significantly higher at ZT4–6 than at ZT18–20 in both WT mice *RORγ^−/−^* mice with fed either a HFD or ND (Figure S1E). However, gluconeogenesis was greatly reduced at ZT4–6 in *RORγ^−/−^* mice compared to WT mice independent of whether the mice were fed a ND or HFD, while little difference in pyruvate tolerance was observed at ZT18–20 between the two genotypes (Figure 2C, S1E). Analysis of the AUC for PTT supported this conclusion (Figure 2D, S1E). *RORγ^−/−^*(HFD) mice also showed a reduced gluconeogenesis at CT4–6, a subjective daytime, under constant darkness (Figure S1B). Together, these observations indicate that loss of RORγ affects pyruvate tolerance particularly at ZT4–6 and support a regulatory role for RORγ in the circadian control of hepatic gluconeogenesis.

To obtain additional evidence that RORγ enhances hepatic gluconeogenesis, we analyzed PTT in *RORγ^−/−^* mice in which RORγ was over-expressed in liver by adenovirus administration. As shown in Figure 2E, gluconeogenesis was significantly increased in mice injected with RORγ-expressing adenovirus compared to mice injected with empty adenovirus. Further support for a role of RORγ in gluconeogenesis was provided by data showing that over-expression of RORγ in *RORγ^−/−^* primary hepatocytes increased glucose production (Figure S2C). Together these results suggested that RORγ modulates insulin resistance and glucose tolerance by regulating hepatic gluconeogenesis.

### Blood insulin and hepatic glycogen levels are reduced in *RORγ^−/−^* mice

**Figure 3:**
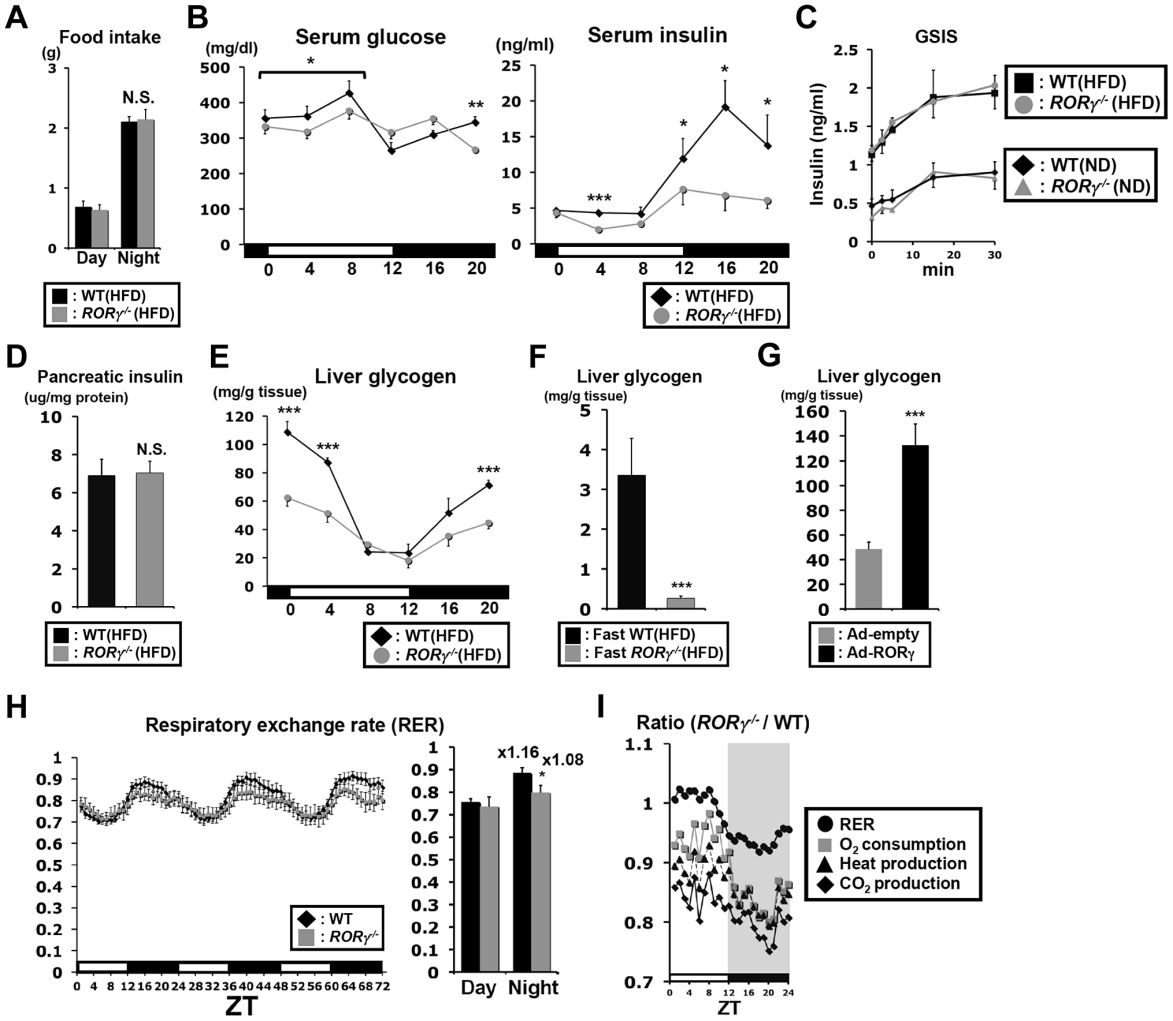
Blood insulin and hepatic glycogen levels are reduced in *RORγ^−/−^* mice. (**A**) Comparison of food consumption between WT(HFD) and *RORγ^−/−^*(HFD) mice (n = 8) during day- and nighttime. (**B**) Serum glucose and insulin levels were analyzed in WT(HFD) and *RORγ^−/−^*(HFD) mice (n = 5) every 4 h over a period of 24 h. (**C**) Comparison of glucose-stimulated insulin secretion (GSIS) in WT and *RORγ^−/−^* mice. Mice were fed either a HFD (n = 5–6) or ND (n = 2–3) and GSIS was analyzed as described in Materials and Methods. (**D**) Analysis of insulin content in pancreas of WT(HFD) and *RORγ^−/−^*(HFD) mice (n = 10–14) collected at ZT16. (**E**) Comparison of glycogen accumulation in livers of WT(HFD) and *RORγ^−/−^*(HFD) mice (n = 5) collected every 4 h over a period of 24 h. (**F**) Analysis of glycogen accumulation in livers from WT(HFD) and *RORγ^−/−^*(HFD) mice (n = 7) collected at ZT4 after 16 h fasting. (**G**) Liver glycogen accumulation was enhanced in liver of *RORγ^−/−^* mice (n = 6) injected with RORγ-expressing adenovirus. (**H, I**) Oxygen consumption (VO_2_), CO_2_ production (VCO_2_), respiratory exchange ratio (RER), and heat production were measured during 3 successive days using metabolic cages and the average in each ZT was plotted as a ratio between *RORγ^−/−^*(ND) and WT(ND) mice (n = 8). Data represent mean ±SEM, * P<0.05, ** P<0.01, *** P<0.001 by ANOVA.

Food intake during daytime and nighttime was not significantly changed in *RORγ^−/−^*(HFD) mice (Figure 3A) and although glucose levels tended to be somewhat lower during daytime, a period in which gluconeogenesis was reduced, serum glucose levels were largely maintained in *RORγ^−/−^*(HFD) mice (Figure 3B). Serum insulin levels in WT mice exhibited a circadian pattern reaching peak levels at ZT16, while insulin levels were significantly lower in both *RORγ^−/−^*(HFD) and *RORγ^−/−^*(ND) mice particularly during ZT12–20 (Figure 3B, S3A). Glucose-stimulated insulin secretion (GSIS) experiments indicated no difference in insulin secretion between WT and *RORγ^−/−^* mice fed with either a ND or HFD (Figure 3C). In addition, little difference was observed in the level of pancreatic insulin at ZT16, the time at which the difference in serum insulin levels was the greatest (Figure 3D). These results suggested that lower serum insulin levels in *RORγ^−/−^* mice were not due to impaired insulin secretion or reduced pancreatic β-cell mass. Moreover, the amount of insulin secretion in response to the same quantity of glucose injected was not changed, suggesting that the reduced insulin level in *RORγ^−/−^* mice is likely due to reduced glucose production.

Glyconeogenesis and glycogenolysis play an important part in glucose homeostasis; 10–20% of hepatic glucose production in mice fasting for 4 h depends on glycogenolysis [27]. Hepatic glycogen reached its highest level at ZT0 and its lowest between ZT8–12 in both WT(HFD) and *RORγ^−/−^*(HFD) mice; however, its peak level was significantly lower in *RORγ^−/−^*(HFD) mice (Figure 3E). After 16 h fasting, the level of hepatic glycogen was dramatically reduced in both WT(HFD) and *RORγ^−/−^*(HFD) mice, but levels remained significantly lower in *RORγ^−/−^*(HFD) mice (Figure 3F). The level of hepatic glycogen was also reduced in *RORγ^−/−^* mice fed with a ND (Figure S3B). Glycogen accumulation was increased in *RORγ^−/−^*(HFD) mice injected with RORγ-expressing adenovirus (Figure 3G), indicating that RORγ positively contributes to hepatic glycogen accumulation. Altogether, these results indicate that *RORγ^−/−^* mice are able to maintain blood glucose levels at lower insulin levels due to reduced hepatic glucose production and possibly reduced glucose uptake by the liver. The latter is consistent with the reduced glycogen accumulation and clamp test data showing that basal HGP/Rd was reduced in *RORγ^−/−^* mice (Figure 2A).

### Loss of RORγ affects energy homeostasis in a diurnal manner

We next examined the behavior activity and energy homeostasis in WT(ND) and *RORγ^−/−^*(ND) mice in relationship to the effect of RORγ on circadian rhythm and hepatic glucose metabolism. No significant difference in total body weight was observed between WT and *RORγ^−/−^* mice fed a ND (Figure S3C). The wheel running test showed that the circadian phase of behavioral activity was not changed in *RORγ^−/−^*(ND) mice consistent with a previous report [12], but peak activity was lower than in WT mice (Figure S3D). Indirect calorimetry showed that oxygen consumption (VO_2_), CO_2_ production (VCO_2_), respiratory exchange ratio (RER), and heat production were significantly lower in *RORγ^−/−^*(ND) mice compared to WT(ND) mice particularly at nighttime (Figure 3H and Figure S3E). Lower RER particularly at nighttime might indicate a preference for fatty acid consumption over glucose for energy production. Plotting of these parameters as a ratio between *RORγ^−/−^*(ND) and WT(ND) mice showed that the largest difference between WT and *RORγ^−/−^* mice occurred around ZT20 (Figure 3I), which corresponds closely to the peak expression of RORγ [10]. These results indicate that the change in glucose metabolism in *RORγ^−/−^* mice is associated with reduced energy expenditure.

### RORγ cistrome is enriched for genes involved in lipid and glucose metabolism

**Figure 4:**
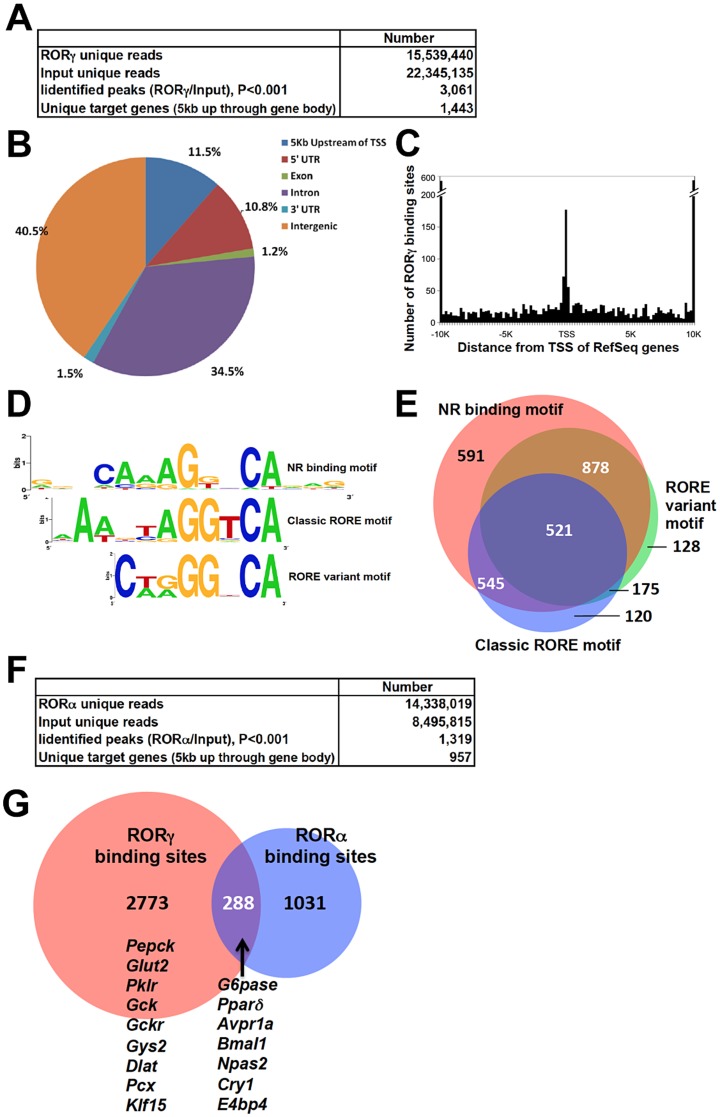
Genome-wide mapping of RORγ and RORα binding sites in mouse liver. (**A**) Summary of ChIP-Seq analysis using an anti-RORγ antibody and mouse hepatic chromatin. The RORγ binding regions were identified by SISSRs, P<0.001. (**B**) Genomic position of RORγ-binding regions on the mouse genome relative to the nearest gene. The promoter is defined as the region up to 5 kb upstream from TSS. (**C**) Distance from the center of each peak identified as a RORγ-binding site to transcriptional start site (TSS) of the nearest gene. (**D**) Motif analysis. *De novo* consensus motif analysis was performed within the RORγ binding sites using MEME program. This analysis identified a classic RORE motif, a DR1-like motif, and a RORE variant motif. (**E**) Venn diagram representing the overlap of the 3 consensus motifs within the RORγ binding regions. (**F**) Summary of ChIP-Seq analysis using an anti-RORα antibody and mouse hepatic chromatin. The RORα binding peaks were identified by SISSRs, P<0.001. (**G**) Venn diagram representing the overlap between the RORγ and RORα binding sites. Examples of genes containing common RORγ and RORα, binding sites and genes containing binding regions unique to RORγ are indicated.

To obtain further insights into the mechanism underlying the regulation of hepatic glucose metabolism by RORγ, we performed ChIP-Seq analysis to determine the genome-wide map of cis-acting targets (cistrome) of RORγ in murine liver at ZT22, a few hours after the peak expression of *RORγ* (Figure S4A) [10]. This analysis identified 3,061 RORγ binding sites (P<0.001) that were localized within intergenic regions (40.5%), introns (34.5%), within a 5 kb region upstream of the transcription start site (TSS)(11.5%), and the 5′UTR (10.8%) (Figure 4A, 4B). Notably, RORγ-binding sites were enriched near the transcription start sites (Figure 4C). *De novo* motif analysis using MEME program identified a classic RORE motif, AGGTCA preceded by an AT-rich region (Figure 4D and 4E) as well as direct repeat 1 (DR1)-like nuclear receptor binding motif and a RORE variant motif. Interestingly, a similar DR1 and variant RORE motifs were recently found within the binding sites of Rev-Erbs [14], [28]. Gene ontology analysis of 1,443 RORγ candidate target genes, defined as those that have one or more detected RORγ binding site within 5 Kb upstream of the TSS and/or within the gene body, indicated that the RORγ cistrome was enriched for genes involved in fatty acid, amino acid, and carbohydrate metabolism (Table 1 and Table S2). Comparison of the ChIP-Seq data with those obtained from our previous microarray analysis [29] indicated that about 23% of the RORγ candidate target genes were differentially expressed between WT and *RORγ^−/−^* liver. CircaDB (http://bioinf.itmat.upenn.edu/circa/) database analysis indicated that about 25% of the RORγ target genes exhibited a rhythmic expression pattern.

**Table 1:** Summary of PANTHER GO analysis for RORγ target genes.

PANTHER Biological Process	Count	*P*-value	FDR
BP00019:Lipid, fatty acid and steroid metabolism	120	2.19E-15	2.79E-12
BP00020:Fatty acid metabolism	44	1.75E-11	2.19E-08
BP00013:Amino acid metabolism	45	4.37E-10	5.49E-07
BP00001:Carbohydrate metabolism	74	3.35E-07	4.21E-04
BP00180:Detoxification	21	1.34E-06	1.68E-03
BP00082:Coenzyme metabolism	13	3.91E-04	4.90E-01
BP00027:Regulation of lipid, fatty acid and steroid	9	1.03E-03	1.280919
BP00272:Phospholipid metabolism	22	1.12E-03	1.400393
BP00022:Fatty acid beta-oxidation	8	1.19E-03	1.482366
BP00292:Other carbon metabolism	15	1.48E-03	1.838969
BP00081:Coenzyme and prosthetic group metabolism	23	1.52E-03	1.896110
BP00011:Monosaccharide metabolism	10	1.55E-03	1.929276
BP00101:Sulfur metabolism	16	3.67E-03	4.515066
BP00223:Angiogenesis	11	3.68E-03	4.529655
BP00017:Amino acid catabolism	10	3.74E-03	4.600846

Because RORα and RORγ bind similar DNA response elements, we examined the degree of functional redundancy between RORγ and RORα in regulating hepatic gene expression by comparing the RORα and RORγ binding sites identified by ChIP-Seq analyses. The specificity of each anti-ROR antibody was confirmed by WB and ChIP assays using chromatin of ROR-deficient mice as a negative control (Figure S4B and S4C). ChIP-Seq analysis identified 1,319 RORα binding sites (P<0.001) and 957 candidate target genes (Figure 4F). Comparison of the RORα and RORγ cistromes revealed that 288 sites, including the ROREs within several clock genes reported previously [10], recruited both RORα and RORγ (Figure 4G and Table S3). Thus, the relatively small overlap indicates that in liver RORα and RORγ exhibit a limited functional redundancy.

### RORγ regulates the circadian expression of glucose metabolic genes

**Figure 5:**
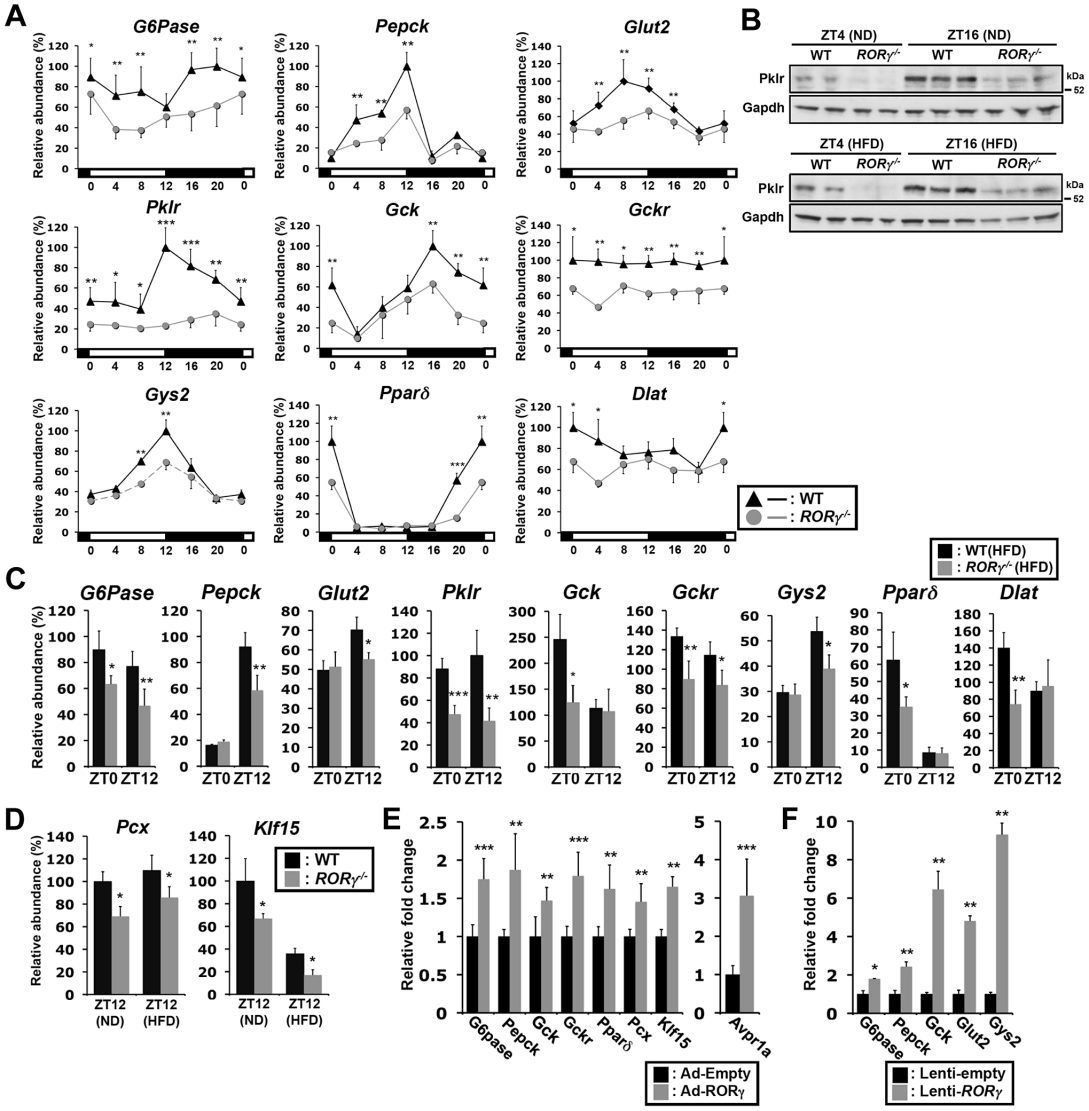
RORγ regulates the circadian expression of genes involved in gluconeogenesis and glycolysis pathways. (**A**) Circadian expression pattern of *G6pase*, *Pepck*, *Glut2*, *Pklr*, *Gck*, *Gckr*, *Gys2*, *Pparδ*, and *Dlat* in liver of WT(ND) and *RORγ^−/−^*(ND) mice (n = 4). RNA was isolated every 4 h over a period of 24 h. (**B**) Pklr protein levels at ZT4 and ZT16 in whole liver lysates prepared from WT and *RORγ^−/−^* mice fed either a ND or HFD (n = 2–3). Pklr was examined by Western blot analysis. (**C**) Differential expression of several metabolic genes in liver of WT(HFD) and *RORγ^−/−^*(HFD) mice collected at ZT0 and ZT12 (n = 5). (**D**) Differential expression of *Pcx* and *Klf15* in WT and *RORγ^−/−^* livers collected at ZT12. (**E**) Adenovirus mediated over-expressing of RORγ in *RORγ^−/−^* liver enhanced the expression of several glucose metabolic genes. (**F**) *G6pase*, *Pepck*, *Gck*, *Glut2*, and *Gys2* expression in primary hepatocytes isolated from *RORγ^−/−^* mice (n = 3) infected with either empty or RORγ lentivirus. Data represent mean ±SD, * P<0.05, ** P<0.01, *** P<0.001 by ANOVA.

Our ChIP-Seq analysis indicated that RORγ is recruited to regulatory regions of several genes implicated in hepatic glucose metabolism, including *G6pase*, *Pepck*, *Glut2*, *Pklr*, *Gck*, *Gckr*, *Gys2*, *Pparδ*, *Pcx* and *Klf15* (Figure 4G, S5). Loss of RORγ resulted in a ZT-dependent decrease in the hepatic expression of most of these genes (Figure 5A–5D) and are consistent with our ChIP-Seq data indicating that their transcription is directly regulated by RORγ. The expression of *G6pase* was repressed in *RORγ^−/−^* liver during most of the circadian cycle, while *Pepck* expression was reduced during ZT4–12; both genes play a key role in gluconeogenesis (Figure 5A). Peak expression of *Gys2*, encoding a rate-limiting enzyme for glycogenesis, and *Pparδ*, which regulates several genes involved in glucose and lipid metabolism [30], was decreased between ZT4–16 and ZT16-4, respectively. The expression of several other gluconeogenic genes, including *Pcx* and *Klf15*, the glucose transporter *Glut2*, and several genes important in the glycolysis pathway, including *Plkr*, *Gck*, and *Gckr*, was also diminished in *RORγ^−/−^* liver (Figure 5A–5D). Decreased expression of these genes was also observed in liver of *RORγ^−/−^* mice fed with a HFD (Figure 5C). Importantly, the loss of RORγ had very little effect on the expression of *Bmal1* and *Clock*, and a limited influence on the expression of *Cry1* and *Rev-Erbα* [10], which all play a critical role in the circadian regulation of lipid/glucose metabolic genes (Figure S6) [10], [12]. These results are consistent with the conclusion that the changes in the circadian pattern of expression of glucose metabolic genes are directly related the loss of RORγ rather than changes in the regulation of clock genes by RORγ.

We further showed that exogenous expression of RORγ in *RORγ^−/−^* liver tissue by adenovirus significantly increased the expression of *G6pase*, *Pepck*, *Gck*, *Gckr*, *Pparδ*, *Pcx*, and *Klf15* as well as the RORγ-target gene, *Avpr1a* (Figure 5E) [10]. Similarly, exogenous expression of RORγ in *RORγ^−/−^* primary hepatocytes significantly activated the expression of several of these genes (Figure 5F). These data are consistent with the conclusion that these genes are positively regulated by RORγ.

To examine whether any of these changes in gene expression translated into alterations in corresponding protein, we analyzed the expression of Pklr, which plays a key role in catalyzing the formation of pyruvate from phosphoenolpyruvate. As shown in Figure 5A and 5B, the level of Pklr protein in WT and *RORγ^−/−^* liver correlated rather well with the level of RNA expression. The levels of Pklr protein and RNA were higher at ZT16 than at ZT4 and clearly repressed in *RORγ^−/−^* liver.

### RORγ activates the target genes through novel ROREs

**Figure 6:**
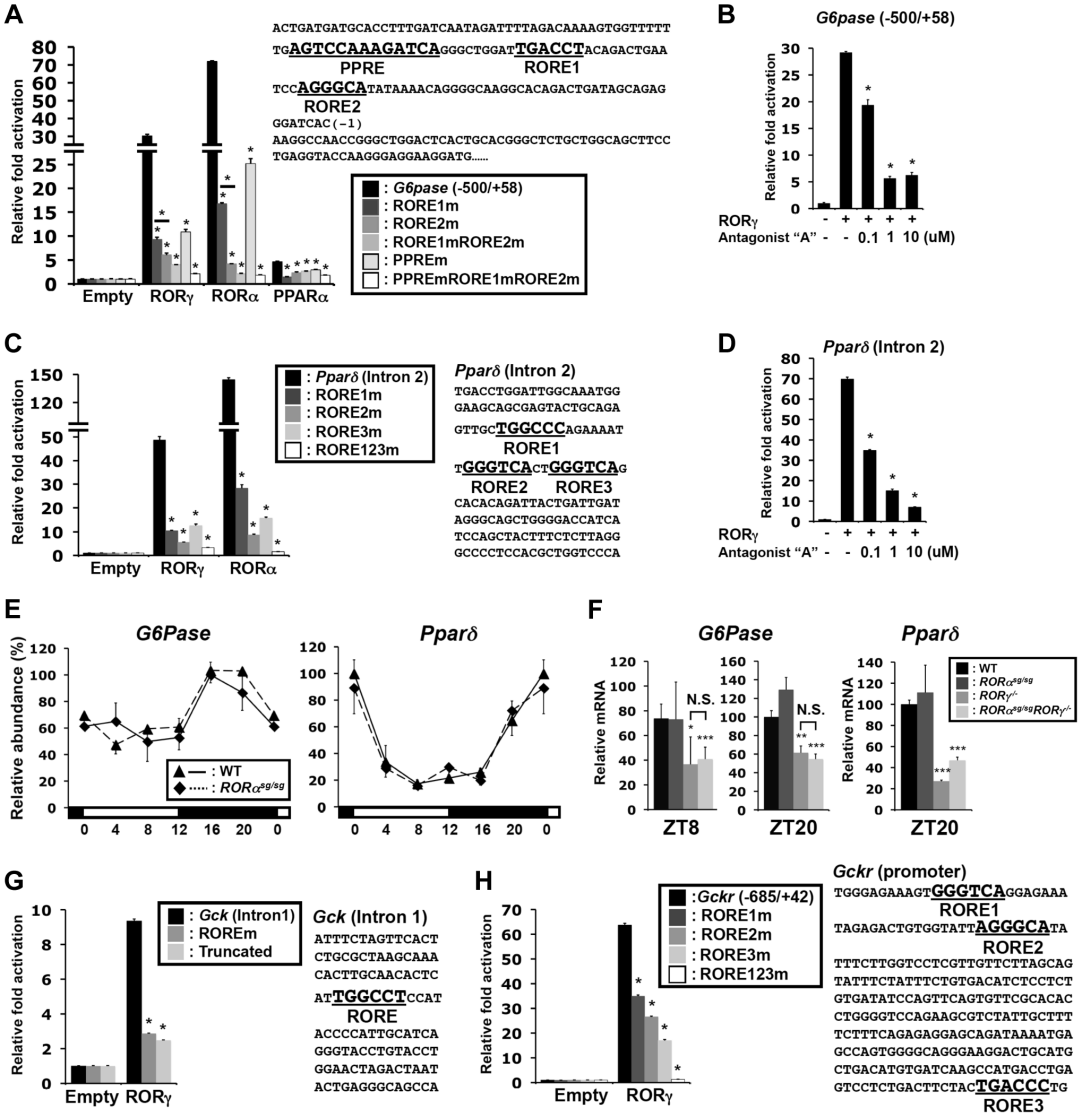
Transcriptional regulation of glucose metabolic genes by RORγ. (**A**) Sequence and activation of the RORγ binding region of the *G6pase*(−500/+58) proximal promoter. The ROREs and PPRE are indicated in bold. Activation of the *G6pase* promoter by RORγ was examined by transfecting Huh-7 cells as indicated with pCMV-β-Gal, pCMV10-3xFlag-RORγ, -RORα or -PPARα (with 10 µM Wy14,643) expression vectors and a pGL4.10 reporter driven by *G6Pase*(−500/+58) or the promoter in which the RORE and PPRE were mutated. Luciferase activities were normalized to the control transfected with the empty expression vector. (**B**) Inhibition of the activation of the *G6pase*(−500/+58) promoter by RORγ-selective antagonist “A”. (**C**) Activation of the *Pparδ* regulatory region by RORγ. Sequence of the RORγ binding region in intron 2 of *Pparδ*. The three potential ROREs are indicated in bold. Huh-7 cells were co-transfected with pCMV-β-Gal, pCMV10-3xFlag-RORγ or -RORα expression vector, and the pGL4.27 reporter plasmid containing the *Pparδ* (intron 2) or the intron in which the ROREs are mutated. (**D**) Inhibition of the activation of the *Pparδ* (intron 2) by the RORγ-selective antagonist. Data represent mean ±SEM, * P<0.05 by ANOVA. (**E**) Loss of RORα does not affect the circadian expression of *G6pase* and *Pparδ* in liver of WT and *RORα^sg/sg^* mice (n = 4). (**F**) Comparison of *G6pase* and *Pparδ* expression in liver collected from WT, *RORα^sg/sg^*, *RORγ^−/−^*, and *RORα^sg/sg^RORγ^−/−^*DKO mice at ZT8 and ZT20. Data represent mean ±SD, * P<0.05, ** P<0.01, *** P<0.001 by ANOVA. (**G**) Huh-7 cells were co-transfected with pGL4.27 in which the reporter was under the control of *Gck* (intron 1) or *Gck* (intron 1) containing a mutated RORE or truncated *Gck* (intron 1) without the RORE. (**H**) Huh-7 cells were co-transfected with pGL4.10 plasmid containing the mouse *Gckr* promoter (−685/+42) or the promoter containing mutated ROREs. Data represent mean ±SEM, * P<0.05 by ANOVA.

Our ChIP-Seq analysis indicated that in liver both RORα and RORγ are recruited to the proximal promoter of *G6pase* and to intron 2 of *Pparδ* (Figure 4G and Figure S5A). ChIP-QPCR analysis showed higher association of RORγ with these regulatory regions at ZT22 compared to ZT10, whereas relatively little recruitment was observed in *RORγ^−/−^* liver at either ZT10 or ZT22 (Figure S5D, S5E). Analysis of the *G6pase* proximal promoter (−500/+58) identified, in addition to a classical RORE (RORE1) [31], a RORE variant motif (RORE2), and a PPAR responsive-element (PPRE) (Figure 6A), which has been reported to mediate the transactivation of *G6pase* by PPARα [32]. Reporter gene analysis showed that both RORγ and RORα were able to highly activate the *G6pase* promoter (Figure 6A), while the RORγ-selective antagonist “A” [10] inhibited the activation by RORγ at concentrations as low as 100 nM (Figure 6B). Mutation of either the RORE1 or RORE2 greatly reduced the activation by RORs. Interestingly, these RORE mutations also inhibited the transcriptional activation of the *G6pase* promoter by PPARα. Inversely, a PPRE mutation significantly reduced the activation by RORs as well as by PPARα, while mutation of both ROREs and PPRE almost totally abolished *G6pase* transactivation (Figure 6A). These observations suggested that RORs and PPARα collectively regulate *G6pase* expression.

The ROR binding region in intron 2 of *Pparδ* contains three putative ROREs, including a variant sequence (Figure 6C). Reporter analysis showed that RORγ and RORα activated the Luc reporter gene driven by this regulatory region about 45- and 140-fold, respectively. Mutation of any of these 3 ROREs strongly reduced the activation of the reporter by RORγ, while the triple mutation almost totally abolished activation. The RORγ antagonist inhibited this activation in a dose-responsive manner (Figure 6D). These results support the conclusion that *Pparδ* transcription is directly regulated by RORγ through these response elements and suggest that the circadian regulation of certain metabolic outputs by RORγ may be in part due to its regulation of *Pparδ* expression.

Although RORα was recruited to the RORE-containing regions of *G6pase* and *Pparδ* (Figure S5D, S5E) and activated the *G6pase* and the *Pparδ* regulatory region in reporter assays, loss of RORα had little effect on the circadian expression of *G6pase* and *Pparδ* (Figure 6E). The expression of these genes in double knockout *RORα^sg/sg^RORγ^−/−^* liver was reduced to a similar degree as in *RORγ^−/−^* liver (Figure 6F). These results suggest that under the conditions tested RORγ rather than RORα, plays a significant role in the hepatic regulation of *G6pase* and *Pparδ in vivo*.

In addition to *G6pase* and *Pparδ*, RORγ was recruited to several other genes important in glucose homeostasis, including intron 1 of *Gck*, the proximal promoter (−685/+42) of *Gckr* (Figure 6G and 6H, Figure S5B), intron 2 of *Glut2*, the promoter of *Gys2*, and the promoter of *Dlat* (Figure S7A). RORγ was able to activate the Luc reporter gene driven by these regulatory regions. Mutation or deletion of the RORE(s) in the *Gck* and *Gckr* regulatory region as well as addition of the RORγ antagonist significantly reduced the activation by RORγ (Figure 6G, 6H, S7B). ChIP-Seq analysis showed that RORα was not associated with these genes, and except for *Gys2*, RORα-deficiency had little effect on the expression of these genes *in vivo* (Figure S7C, S7D). Together, these results support the conclusion that RORγ directly regulates the transcription of a series of genes important in glucose metabolism and homeostasis.

### Liver-specific RORγ^−/−^ mice exhibit reduced gluconeogenesis and improved insulin sensitivity

**Figure 7:**
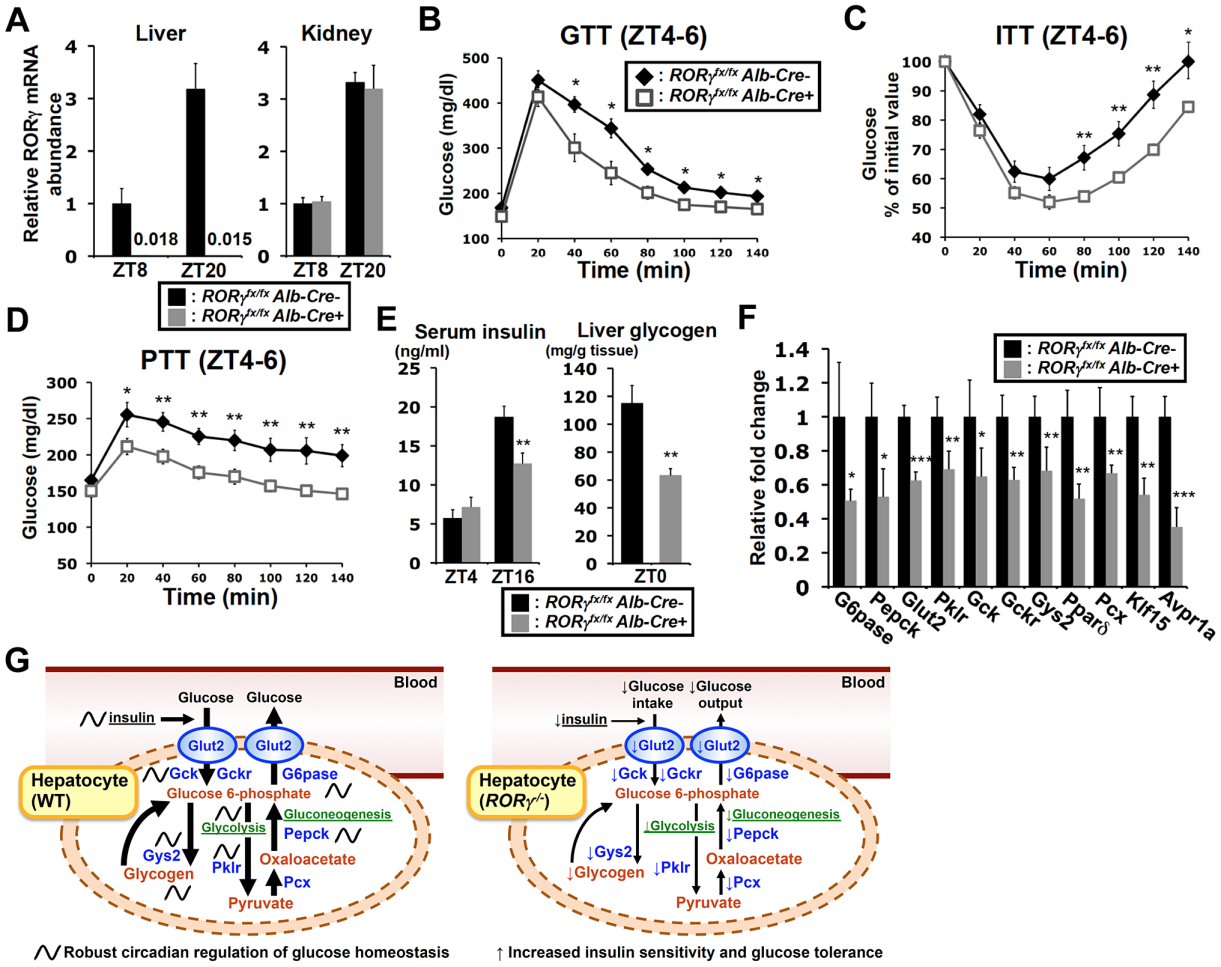
Liver-specific RORγ deficient mice exhibit improved insulin sensitivity and reduced gluconeogenesis. (**A**) *RORγ* expression in liver and kidney collected from *RORγ^fx/fx^Alb-Cre^+^* and -*Alb-Cre^−^* mice at ZT8 and ZT20 (n = 4–5). GTT (**B**), ITT (**C**), and PTT (**D**) were examined during ZT4–6 in *RORγ^fx/fx^Alb-Cre^+^* and -*Alb-Cre^−^* mice fed with a HFD (n = 7–11). (**E**) Serum insulin levels were measured at ZT4 (n = 8) and ZT16 (n = 15–16) in *RORγ^fx/fx^Alb-Cre^+^* and -*Alb-Cre^−^* mice on a HFD. Hepatic glycogen was measured at ZT0. Data represent mean ±SEM, * P<0.05, ** P<0.01 by ANOVA. (**F**) The expression of a series of glucose metabolic genes was analyzed in the liver collected at ZT8 or ZT20 (n = 4–5). Data represent mean ±SD, * P<0.05, ** P<0.01, *** P<0.001 by ANOVA. (**G**) RORγ coordinates the regulation of circadian rhythm, hepatic glucose metabolism, and insulin sensitivity. Genome-wide cistromic profiling and promoter analysis revealed that RORγ is targeting and regulating a number of metabolic genes critical in the control of glycolysis, gluconeogenesis and glycogenesis pathways. The loss of RORγ in hepatocytes reduces the expression of these genes and hepatic gluconeogenesis in a diurnal time-dependent manner that results in improved insulin sensitivity. Due to reduced hepatic glucose production, *RORγ^−/−^* mice may require less insulin than WT mice to maintain blood glucose levels. A decrease in glucose uptake due to lower insulin levels as well as reduced Gys2 expression may in part be responsible for the reduced accumulation of liver glycogen. Our study supports the model that the circadian regulation of several glucose metabolic genes by RORγ in liver is linked to its circadian control of gluconeogenesis, insulin sensitivity, and glucose tolerance and is consistent with the idea that RORγ functions as a positive regulator of gluconeogenesis and is positively linked to increased risk for type 2 diabetes.

To determine whether the effects on hepatic glucose metabolism were based on the hepatocyte-specific loss of RORγ function rather than loss of RORγ in other metabolic tissues or immune cells, we analyzed liver-specific RORγ-deficient (*RORγ^fx/fx^Alb-Cre^+^*) mice. Our data confirmed that *RORγ* expression was completely lost in the liver of *RORγ^fx/fx^Alb-Cre^+^* mice and was not changed in the kidney (Figure 7A). ITT, GTT, and PTT analysis showed that, as demonstrated for the RORγ ubiquitous knockout mice, *RORγ^fx/fx^Alb-Cre^+^*(HFD) mice exhibited a greater glucose tolerance, were more responsive to insulin, and showed reduced gluconeogenesis, respectively (Figure 7B–7D). Moreover, as in *RORγ^−/−^* mice, the blood insulin concentration at ZT16 was significantly reduced in *RORγ^fx/fx^Alb-Cre^+^*(HFD) mice and so was the peak accumulation of hepatic glycogen at ZT0 (Figure 7E). Moreover, gene expression analysis showed that the hepatic expression of a series of RORγ target genes important in glucose metabolism, including *G6pase* and *Pparδ*, were also decreased in *RORγ^fx/fx^Alb-Cre^+^* mice as seen in *RORγ* ^−/−^ mice (Figure 7F). Together, these observations suggest that the changes in hepatic glucose metabolism are related directly to the loss of RORγ function in the liver and support the conclusion that RORγ directly contributes to the regulation of hepatic gluconeogenesis and glucose metabolism.

## Discussion

In this study, we identify a novel function for RORγ in the daily regulation of hepatic glucose metabolism and insulin sensitivity. Our results demonstrate that at ZT4–6 *RORγ^−/−^* mice are significantly more insulin sensitive and glucose tolerant than WT mice, while there was a smaller difference between the two strains at ZT18–20. The euglycemic clamp test revealed that hepatic glucose production was considerably reduced in *RORγ^−/−^* mice (Figure 2A). This was supported by PTT data showing that the conversion of exogenously administered pyruvate to glucose was significantly lower in *RORγ^−/−^* mice particularly at ZT4–6 (Figure 2C). Inversely, ectopic RORγ expression in *RORγ* ^−/−^ liver tissue or primary hepatocytes increased glucose production (Figure 2E, S2C). Our ITT and PTT data indicate that the regulation of glucose metabolism by RORγ is also functional at subjective daytime, CT4–6, under constant darkness (Figure S1A, S1B). Together, these observations demonstrate that gluconeogenesis is less efficient in *RORγ^−/−^* liver and support the conclusion that RORγ is an important positive regulator of hepatic gluconeogenesis and insulin sensitivity particularly during early daytime.

The regulation of glucose metabolism is complex and not only depends on hepatic metabolism, but also involves control of metabolic pathways in other tissues in which RORγ is expressed, such as adipose and skeletal muscle. It also involves certain regions of the brain, including the SCN and the hypothalamus, which are implicated in the regulation of the central circadian clock and appetite, respectively [16]–[18]. However, in contrast to RORα and RORβ, RORγ is not or very poorly expressed in the SCN, hypothalamus or other parts of the brain [11], [33]. Therefore, it appears unlikely that the brain plays a major role in the phenotypic changes observed in *RORγ^−/−^* mice. In addition, many of the changes in *RORγ^−/−^* mice, including the reduction in glucose metabolic gene expression, were also observed in liver-specific *RORγ*-deficient mice, indicating that these effects are directly related to the loss of RORγ in hepatocytes and separate from the loss of RORγ in other metabolic tissues (Figure 7F).

Since RORγ functions as a transcription factor, the reduced gluconeogenesis in RORγ-deficient mice must involve alterations in the transcription of RORγ target genes. *De novo* motif analysis of the RORγ cistrome identified, in addition to the classic RORE, two variant RORE-like motifs. The variant ROREs appear to allow a greater diversity in ROR binding than expected from the *in vitro* binding assays [34], [35]. A greater promiscuity in *in vivo* DNA binding has also been observed for other nuclear receptors, and might be due to promoter context, chromatin structure, and histone modifications. Gene ontology analysis showed that many of the potential RORγ-target genes are linked to metabolic pathways (Table 1 and Table S2), including glucose homeostasis (*e.g.*, *G6pase*, *Pepck*, *Pklr*, *Pparδ*, *Gck*, *Gckr*, *Glut2*, *Gys2*, *Dlat*, *Pcx*, and *Klf15*). Analysis of their rhythmic pattern of expression demonstrated that RORγ deletion reduced peak expression of most of these genes, without affecting their phase. Regulation of these genes by RORγ was supported by data showing that exogenous expression of RORγ in *RORγ^−/−^* liver and primary hepatocytes significantly enhanced their level of expression (Figure 5E, 5F). Promoter and mutation analysis demonstrated that RORγ was able to activate several of the RORE-containing promoters, while mutation of either the classic or variant ROREs significantly reduced this activation by RORγ indicating that these motifs are functional. The RORγ-mediated promoter activation was further supported by data showing that treatment with a RORγ-selective antagonist considerably inhibited this activation (Figure 6B, 6D, S7B). Our RORγ cistrome data together with the mRNA expression and promoter analysis support the model that in murine liver, RORγ positively regulates the expression of a series of glucose metabolic genes directly through RORE binding. The reduced peak expression of several key metabolic genes, including G6pase and Pepck, which are critical for gluconeogenesis, the glucose transporter Glut2, and several genes important in the glycolysis pathway, including *Plkr*, *Gck*, and *Gckr*, likely contribute to the reduced glucose uptake, the less efficient gluconeogenesis and the lower glycogen accumulation observed in RORγ deficient liver.

In addition to RORγ, glucose metabolism is under the control of a number of other transcription factors. Although loss of RORγ reduced peak expression of several glucose metabolic genes, most of these genes still exhibited a substantial rhythmic pattern of expression, indicating that additional factors are involved. For example, analysis of the *G6pase* promoter showed that in addition to the classic and variant RORE proximal promoter, it contained a PPRE (Figure 6A), which has been reported to mediate the transactivation of *G6pase* by PPARα [32]. Mutation of either the ROREs or PPRE caused a significant reduction in the activation of this promoter suggesting that RORγ and PPARα cooperatively regulate *G6pase*. Although comparison of the RORα and RORγ cistromes indicated that RORα and RORγ have largely distinct functions, there was a 10% overlap in target genes that included several glucose metabolic genes, such as *G6pase* and *Pparδ* (Figure S5). However, in contrast to *RORγ^−/−^* mice, loss of RORα did not affect the expression of *G6pase* or *Pparδ* (Figure 6E, 6F) suggesting that under the conditions tested these genes are regulated by RORγ rather than RORα.

Although several studies have demonstrated a role for Bmal1 and Clock in the regulation of several metabolic genes and shown that RORγ is recruited to ROREs in *Clock* and *Bmal1*, the loss of RORγ had little effect on the hepatic expression of *Bmal1* and *Clock* (Figure S6) [8], [10]. These observations suggest that changes in glucose metabolic genes in *RORγ^−/−^* liver are not due to changes in *Clock* or *Bmal1* expression and are consistent with the hypothesis that RORγ regulates these genes downstream of the clock machinery. However, cistrome analysis has shown that Bmal1 can also be recruited to certain glucose metabolic genes, such as *G6pase*, suggesting that Bmal1 in conjunction with RORγ positively regulates the expression of these genes. In addition, RORγ might cause changes in chromatin structure and as such influences the recruitment of Bmal1 or Clock to common target genes. The Rev-Erb nuclear receptors also play a critical regulatory role in the robust oscillation of circadian expression of a number genes [14]. RORs and Rev-Erb receptors can interfere with each other's activity by competing for RORE binding [10]. Despite the modest reduction in peak expression of Rev-Erbα in *RORγ* ^−/−^ liver (Figure S6), which should result in increased target gene expression, the loss of RORγ may reduce the competition with Rev-Erbα for RORE binding and as a consequence increase the repression of gene transcription by Rev-Erbα. A more comprehensive comparison between the cistrome of RORs, clock proteins, and Rev-Erbs is needed to provide further insights into the crosstalk between these transcription factors.

Although insulin levels were significantly lower in *RORγ^−/−^* mice, blood glucose levels were largely maintained (Figure 3B). At daytime, hepatic glucose production is less efficient in the knockout mice and consistent with this, blood insulin level was significantly reduced at ZT4. We hypothesize that insulin sensitivity in *RORγ^−/−^* mice is also improved during nighttime due to reduced hepatic glucose production, which as a consequence would require less insulin to maintain blood glucose level and explain the lower level of blood insulin in *RORγ^−/−^* mice. This is supported by AUC analysis for ITT, which indicates that also at nighttime insulin sensitivity was significantly better in *RORγ^−/−^* mice (Figure 1B). When mice eat during nighttime, more insulin is required to maintain blood glucose levels and this may account for the greater difference in blood insulin level compared to the difference at daytime. The observation that the PTT indicated little changed in gluconeogenesis efficiency at nighttime may be related to the fact that the PPT determines the efficiency of the gluconeogenesis pathway by measuring the formation of glucose from pyruvate after exogenous pyruvate injection, which is not a total reflection of all the pathways involved in the regulation of hepatic gluconeogenesis *in vivo* because pyruvate for gluconeogenesis can be supplied by other metabolic pathways.

A lower RER is considered to indicate that fat is increasingly preferred as a fuel source, whereas a higher RER is indicative for an increased use of carbohydrates. Thus, the lower RER observed at daytime in both WT and knockout mice indicates a greater preference for fatty acid consumption over glucose compared to nighttime (Figure 3H), while the lower nighttime RER levels in *RORγ^−/−^* mice compared to WT mice indicate a greater preference for fatty acid consumption over glucose. The latter is likely related to reduced glucose production and reduced glucose uptake in RORγ knockout liver. Our data show that hepatic glycogen accumulation was reduced in RORγ knockout mice during ZT16-0 clearly indicating that loss of RORγ also affects glucose homeostasis at nighttime. This reduction in glycogen is likely due a reduced glucose uptake, which correlate with the lower levels of blood insulin in RORγ knockout mice (Figure 3B and 3E). Further analyses will be needed to precisely understand the precise interrelationships between various transcription factors, their diurnal regulation of various metabolic pathways and glucose and energy homeostasis.

In summary, our study identifies a novel function for RORγ in the regulation of gluconeogenesis and insulin resistance. Our data are consistent with the model in which RORγ directly regulates the expression of glucose metabolic genes in the liver downstream of the hepatic circadian clock, thereby enhancing gluconeogenesis, and decreasing insulin sensitivity and glucose tolerance (Figure 7G). The temporal organization of tissue metabolism is coordinated by reciprocal crosstalk between the core clock machinery and key metabolic enzymes and transcription factors. Our study indicates that RORγ is a novel important participant in this crosstalk. The improved insulin sensitivity and glucose tolerance observed in RORγ-deficient mice suggest that the loss of RORγ might be beneficial in controlling glucose homeostasis and in the management of metabolic diseases. This is supported by recent studies showing that in human patients the level of *RORγ* expression positively correlates with insulin resistance [20], [21]. The inhibition of the activation of several glucose metabolic gene promoters by an RORγ-selective antagonist, thereby mimicking the effects in *RORγ^−/−^* liver, suggests that such antagonists might provide a novel therapeutic strategy in the management of insulin resistance and type 2 diabetes.

## Materials and Methods

### Experimental animals

Heterozygous C57BL/6 staggerer (*RORα^+/sg^*) were obtained from the Jackson Laboratories (Bar Harbor, ME). *RORγ^−/−^* and *RORα^sg/sg^RORγ^−/−^* double knockout (DKO) mice were described previously [10], [36]. Liver-specific RORγ knockout mice, referred to as *RORγ^fx/fx^Alb-Cre^+^*, were generated by crossing B6(Cg)-*Rorc^tm3Litt^*/J (*RORγ^fx/fx^*) with B6.Cg-Tg(*Alb-cre*)21Mgn/J transgenic mice (Jackson Laboratories). Mice were supplied *ad libitum* with NIH-A31 formula (normal diet, ND) and water, and maintained at 23°C on a constant 12 h light∶12 h dark cycle. Two month-old male mice were fed with a high fat diet (40% kcal fat) (HFD: D12079B Research Diets Inc., New Brunswick, NJ) for 6 weeks. Littermate wild type (WT) mice were used as controls. All animal protocols followed the guidelines outlined by the NIH Guide for the Care and Use of Laboratory Animals and were approved by the Institutional Animal Care and Use Committee at the NIEHS.

### Glucose tolerance test (GTT), insulin tolerance test (ITT), and pyruvate tolerance test (PTT)

After 16 h fasting, WT and *RORγ^−/−^* mice (n = 8–10) fed a ND or HFD for 6 weeks were injected intraperitoneally with glucose (2 g/kg), insulin (0.75 U/kg) (Eli Lilly, Indianapolis, IN) or sodium pyruvate (2 g/kg) (Sigma-Aldrich) at ZT4 or ZT18. The blood glucose was measured every 20 min for up to 140 min with glucose test strips (Nova Biomedical, Waltham, MA). These tests were performed in the same way using *RORγ^fx/fx^Alb-Cre^+^* and *RORγ^fx/fx^Alb-Cre^−^* mice (n = 11) fed a HFD. ITT and PTT were also performed under red light at CT4 after WT(HFD) and *RORγ^−/−^*(HFD) mice (n = 12) were kept for 1 day under constant darkness. Total AUC (Area under the curve) was calculated by the trapezoid rule. Two-way ANOVA was performed using GraphPad PRISM software.

### Western blot analysis

To evaluate insulin signaling, liver, BAT, WAT, and skeletal muscle were isolated from fasting WT(HFD) and mice *RORγ^−/−^*(HFD) mice 30 min after injection with either 0.75 U/kg insulin or PBS. Protein from these tissues was extracted with lysis buffer (25 mM Tris-HCl pH 7.6, 150 mM NaCl, 1% Nonidet P-40, 1% sodium deoxycholate, 0.1% SDS). In a separate experiment, primary hepatocytes isolated from WT and *RORγ^−/−^* mice were treated with 20 nM insulin in serum-free 199 medium (Sigma-Aldrich) for 10 min. Phosphorylated Akt (Ser473) and whole Akt proteins were detected by Western blot analysis with antibodies 7408 and 7102 from Cell Signaling Technology. Pklr and Gapdh were detected in liver lysates from WT and *RORγ^−/−^* mice (n = 3) at ZT4 and ZT16 by Western blot analysis with anti-Pklr (22456-1-AP, Proteintech Group Inc., Chicago, IL, USA) and anti-Gapdh (Cell Signaling Technology) antibodies.

### Hyperinsulinemic-euglycemic clamp test

WT and *RORγ^−/−^* mice (n = 5) fed a HFD for 6 weeks underwent surgery under anesthesia to attach catheters to the jugular vein and carotid artery. Mice were left at least 2 days to recover. After a 3.5 h fasting, the basal rates of glucose turnover were measured by continuous infusion of HPLC-purified D-[3-^3^H] glucose (0.05 µCi/min) (Perkin Elmer, Boston, MA) for 90 minutes following a bolus of 1 µCi. Blood samples (about 40 µl) were taken from the carotid artery catheter at 75 and 85 min after the infusion to determine the plasma [3-^3^H] glucose concentration. Subsequently the hyperinsulinemic euglycemic clamp test was performed for 120 min in conscious, restrained mice. Human insulin (HumulinR, Eli Lilly) was infused at a constant rate (30 mU/kg/min) through the end of the experiment following a bolus of 90 mU/kg/min for 3 min. Glucose was measured every 10 min in blood from tail vein with glucose test strips. The glucose concentration was maintained at 110–130 mg/dl by a variable rate of 20% glucose infusion under a continuous infusion of [3-^3^H] glucose (0.1 µCi/min). Blood samples (about 40 µl) were taken from the carotid artery catheter every 10 min during the last 40 min. [^3^H]-glucose was used to trace hepatic glucose production and glucose turnover. The experiment was performed during daytime at ZT2–9.

For the determination of the plasma ^3^H-glucose concentration, plasma samples were deproteinized with 0.3 N Ba(OH)_2_ and ZnSO_4_ and dried to remove ^3^H_2_O before the radioactivity was measured in a liquid scintillation counter. Basal hepatic glucose production (Basal HGP) was calculated as the ratio of the preclamp [^3^H]-glucose infusion rate (GIR) (dpm/min) to the specific activity of plasma glucose. Clamp whole-body glucose disappearance (Rd) was calculated as the ratio of the clamp [3-^3^H] GIR (dpm/min) to the specific activity of plasma glucose. Clamp glucose production (Clamp HGP) was determined by subtracting the average GIR in the last 40 min from the Rd.

### Preparation and injection of recombinant adenovirus

Recombinant adenoviruses were generated using the AdEasy adenoviral system (Agilent Technologies, Palo Alto, CA). Full-length *RORγ1* cDNA was inserted to pShuttle-IRES-hrGFP-1 vector, and co-transformed with pAdEasy-1 in BJ5183-AD-1 bacteria by electroporation. The recombinant adenovirus plasmid was then transfected in AD-293 cells. The amplified adenoviruses were purified and concentrated by cesium chloride density gradient centrifugation. The empty control and RORγ expressing adenoviruses were injected into the retro-orbital sinus of *RORγ^−/−^*(HFD) mice (n = 6–7). Pyruvate tolerance test was performed 4 days later and after an additional four days, liver was collected at ZT8 to analyze glycogen accumulation and gene expression.

### Primary hepatocyte isolation and glucose production assay

Hepatocytes from 2 month-old WT and *RORγ^−/−^* mouse were isolated with a Hepatocyte Isolation System (Worthington Biochemical Corporation, New Jersey, USA) according to the manufacturer's instructions. Primary hepatocytes were cultured in collagen-coated dishes with Medium 199 supplemented with 100 nM dexamethasone, 1 nM insulin, 10 nM triiodothyronine, 5% fetal bovine serum, and penicillin/streptomycin. After 8–12 h, cells were infected with empty lentivirus pLVX-mCherry-N1 or RORγ1-expressing lentivirus. 24 h later cells were washed twice in PBS and then incubated in serum-free medium 199 in the presence or absence of 100 nM insulin or 100 nM glucagon (Sigma-Aldrich) for 6 h before RNA was isolated. Glucose production was measured with a glucose production buffer (glucose/phenol red-free DMEM (Sigma-Aldrich), 1 mM lactose, 2 mM sodium pyruvate) in *RORγ^−/−^* hepatocyte infected with lentivirus for each empty and RORγ expression (n = 3). Glucose in the medium was measured with a Glucose assay kit (Sigma-Aldrich).

### Insulin, liver glycogen, pyruvate measurement

Serum and liver samples were collected from WT and *RORγ^−/−^* mice on a HFD (n≥5) every 4 h over a period of 24 h. Serum insulin was measured by a sandwich ELISA with a Rat/Mouse Insulin ELISA kit (EZRMI-13K, Millipore). Glucose stimulated insulin secretion (GSIS) was measured at ZT4 in WT and *RORγ^−/−^* mice on a HFD (n = 5–6) or ND (n = 2–3). Serum was collected at 2.5, 5, 15, and 30 min after intraperitoneal injection of glucose (2 g/kg). Pancreatic insulin was determined by rapidly removing the pancreas from WT and *RORγ^−/−^* mice (n = 10–14) on a HFD. Pancreas was then homogenized and extracted overnight with acid-ethanol at −20°C. Insulin in the extracts was measured with the insulin ELISA kit. Insulin was normalized by total pancreatic protein. Glycogen extracted from liver with 30% KOH at 100°C for 2 h followed by precipitation by ethanol, was measured with a Glycogen Assay Kit (BioVison Inc., Mountain View, CA).

### LabMaster metabolic analysis

To analyze metabolic parameters including oxygen consumption, CO_2_ production, respiratory exchange ratio, heat production, and food/water consumptions were measured in WT and *RORγ^−/−^* mice (n = 8) with a LabMaster system (TSE systems Inc., Chesterfield, MO) during 4 successive days.

### Chromatin immunoprecipitation (ChIP) assay

The ChIP assay was performed using a ChIP assay kit from Millipore (Billerica, MA) according to the manufacturer's protocol with minor modifications as described previously [10]. Briefly, livers collected from WT, *RORα^sg/sg^*, and *RORγ^−/−^* mice at ZT10 and ZT22 were homogenized with a polytron PT 3000 (Brinkmann Instruments) and crosslinked by 1% formaldehyde for 10 min at room temperature. After a wash in PBS, an aliquot of the crosslinked chromatin was sonicated and incubated overnight with an anti-RORα or anti-RORγ antibody [10] generated against amino acids 129–231 and 121–213 in mouse RORγ1 and RORα4, respectively. After incubation with protein G agarose beads for 2 h, DNA-protein complexes were eluted. The crosslinks were reversed by overnight incubation at 65°C in the presence of 25 mM NaCl, digested with RNase A and proteinase K, and then the ChIPed-DNA was purified. The amount of ChIPed-DNA relative to each input DNA was determined by QPCR. All QPCR reactions were carried out in triplicate. Sequences of primers for ChIP-QPCR are listed in Table S4.

### ChIP-Seq data analysis

ChIPed-DNA and input DNA as a control were prepared using RORγ- and RORα-specific antibodies as described previously [10]. ChIP-Seq analysis was performed by the NIH Intramural Sequencing Center and data were analyzed as reported previously [37]. The sequencing reads were obtained from base-calling of Illumina Genome Analyzer. The wiggle-formatted alignment results were visualized on UCSC Genome Browser using mouse mm9 reference genome. SISSRs (Site Identification from Short Sequence Reads) were used for identification of significant RORγ and RORα binding sites (P<0.001) that have enriched reads in each ChIPed-DNA versus input control across the whole genome [38]. The distance from each ROR peak to the nearest transcriptional start sites was determined using custom scripts. *De novo* consensus motif search within ROR binding sites was performed using MEME. ChIP-Seq data was compared with gene expression data using Kolmogorov-Smirnov (KS) plot. Gene ontology analysis was performed using the NIH Database for Annotation, Visualization, and Integrated Discovery (DAVID) online web-server, and based on PANTHER Biological process definitions.

### QRT-PCR analysis

To quantify gene expression during circadian time, liver tissues were collected from WT, *RORγ^−/−^*, and *RORα^sg/sg^* mice every 4 h over a period of 24 h, processed overnight in RNA*later* solution (Ambion, Austin, TX) at 4°C, and then stored at −80°C until use. Tissues were then homogenized with a Polytron PT-3000 (Brinkmann Instruments, Westbury, NY). Liver tissues were also collected from *RORα^sg/sg^RORγ^−/−^* DKO mice and littermate control WT mice, and *RORγ^fx/fx^Alb-Cre^+^* and *RORγ^fx/fx^Alb-Cre^−^* mice at zeitgeber time (ZT) 8 and ZT20. RNA was then extracted using a QIAshredder column and RNeasy Mini kit (Qiagen, Valencia, CA) according to the manufacturer's instructions. The RNA was reverse-transcribed using a High-Capacity cDNA Archive Kit (Applied Biosystems). QPCR analysis was performed using SYBR Green I (Applied Biosystems, Foster City, CA). The reactions were carried out in triplicate using 20 ng of cDNA and the following conditions: 10 min at 95°C, followed by 40 cycles of 15 sec at 95°C and 60 sec at 60°C. The results were normalized by the amount of *Gapdh* mRNA. Primer sequences are listed in Table S4.

### Reporter gene assay

The promoter or intron region of mouse *G6Pase* (promoter; −500/+58), *Pparδ* (intron 2; +46417/+46987), *Gck* (intron 1; +29709/+30121), *Gckr* (promoter; −685/+42), *Glut2* (intron 2; +16294/+16805), *Gys2* (promoter; −256/+345), and *Dlat* (promoter; −1151/+22) genes was amplified using mouse genomic DNA (Promega, Madison, WI) and cloned into either the promoter-less reporter plasmid pGL4.10 or pGL4.27 containing a minimal promoter (Promega, Madison, WI). Point mutations in ROREs and PPREs were generated using a Quickchange Site-Directed Mutagenesis Kit (Stratagene, La Jolla, CA). Human hepatoma Huh-7 cells were co-transfected with the indicated pGL4 reporter plasmid, pCMV-β-Gal, and p3xFlag-CMV10-RORγ, –RORα, -Rev-Erbα, or -PPARα expression plasmids using lipofectamine 2000 (Invitrogen, Carlsbad, CA). After 24 h incubation, the luciferase and β-galactosidase activities were measured with a Luciferase Assay Substrate kit (Promega) and Luminescent β-galactosidase Detection Kit II (Clontech). All transfections were performed in triplicate and repeated at least twice. In certain experiments cells were treated for 24 h with a RORγ-selective antagonist “A”, (R)-N-(1-((4-methoxy-phenyl)sulfonyl)-4-methyl-1,2,3,4-tetrahydroquinolin-7-yl)-2,4,6-trimethylbenzene-sulfonamide provided by Dr. Veronique Birault (GlaxoSmithKline) [10] or with the selective PPARα antagonist, Wy14,643 (10 µM; Sigma-Aldrich) as indicated.

## Supporting Information

Figure S1 *RORγ^−/−^*(HFD) mice exhibited improved insulin sensitivity and hepatic gluconeogenesis under ZT-free condition (constant darkness). ITT (A) and PTT (B) were performed during CT4–6, a subjective daytime, in WT(HFD) and *RORγ^−/−^*(HFD) mice (n = 11–12). Mice were kept under constant darkness for 1 day before the start of the experiments. Bar graphs show AUC for ITT and PTT. (C–E) *RORγ^−/−^* mice fed a ND exhibited improved insulin sensitivity and glucose tolerance. ITT, GTT, and PTT were performed during ZT4–6 and ZT18–20 in WT(ND) and *RORγ^−/−^*(ND) mice (n = 7–9). Bar graphs show AUC for ITT, GTT and PTT. Data represent mean ±SEM, * P<0.05, ** P<0.01, *** P<0.001 by ANOVA. Total AUC for ITT, GTT and PTT in (C–E) was also evaluated by 2-way ANOVA (ITT: Time period: P = 0.1234, Genotype: P = 0.0045; GTT: Time period: P = 0.8575, Genotype: P = 0.0018; PTT: Time period: P = 0.0623, Genotype: P = 0.0472; not shown).
(TIF)

Figure S2 Blood glucose level and GIR during insulin clamp test. (A) Blood glucose levels were measured every 10 min for 2 h during the insulin clamp test. The levels were maintained between 110 to 130 mg/dl. (B) Average GIR during the insulin clamp test. (C) Glucose production in primary *RORγ^−/−^* hepatocytes infected with empty or RORγ lentivirus (n = 3). Data represent mean ±SEM, * P<0.05, ** P<0.01, *** P<0.001 by ANOVA.
(TIF)

Figure S3 *ROR^−/−^* mice exhibit reduced energy expenditure at nighttime. (A) Serum insulin levels were compared between WT(ND) and *RORγ^−/−^* (ND) mice (n = 5) at ZT16. (B) Glycogen levels were analyzed in livers from WT(ND), *RORα^sg/sg^*(ND), *RORγ^−/−^*(ND), and *RORα^sg/sg^RORγ^−/−^*(ND) mice (n = 4) collected at ZT2. Serum insulin and hepatic glycogen levels are reduced in *RORγ^−/−^*(ND) mice. Data represent mean ±SEM, * P<0.05, ** P<0.01 by ANOVA. (C) Body weights were not significantly different between WT(ND) and *RORγ^−/−^*(ND) mice. (D) Locomoter activity in WT(ND) and *RORγ^−/−^*(ND) mice (n = 9–11) was evaluated by the wheel running test. (E) Oxygen consumption (VO_2_), CO_2_ production (VCO_2_), and heat production in WT(ND) (black bars and lines) and *RORγ^/−^*(ND) (grey bars and lines) mice (n = 8) were measured during 3 successive days using metabolic cages. The mice were kept under 12 h/12 h light/dark cycles. The numbers indicate fold-increase between day and night in each WT and *RORγ^/−^* mice. Data represent mean ±SEM, * P<0.05, ** P<0.01, *** P<0.001 by ANOVA.
(TIF)

Figure S4 Specificity of anti-ROR antibodies used in ChIP-Seq analysis. (A) *RORα* and *RORγ* mRNA expression were compared by QPCR in the liver collected from WT mice at ZT8 and ZT20 (n = 4). (B) Western blot analysis was performed using lysates prepared from HEK293 cells over-expressing RORα or RORγ and antibodies against RORα or RORγ. (C) ChIP-QPCR was performed using each anti-ROR antibody and chromatin prepared from livers of WT, *RORα^sg/sg^*, and *RORγ^−/−^* mice (n = 4) at ZT22. Amplification of *Bmal1* RORE and *Gapdh* was used as a positive and negative control, respectively. Data represent mean ±SEM, *** P<0.001 by ANOVA.
(TIF)

Figure S5 Mapping of RORγ or RORα binding sites to several gene loci in mouse liver. (A, B) UCSC Genome Browser tracks derived from RORγ and RORα ChIP-Seq data are shown in *G6pase* and *Pparδ* genes (A), *Glut2*, *Pklr*, *Gck*, *Gckr*, *Gys2*, *Pcx*, *Klf15*, and *Dlat* genes (B). (C–E) To confirm ROR binding to *Pepck*(−486/−364) (C), *G6pase*(−500/+58) (D), and *Pparδ*(*intron2*) (E) ChIP-QPCR was performed using anti-RORγ or -RORα antibody and chromatin prepared from the liver of WT, *RORγ^−/−^* or *RORα^sg/sg^* mice (n = 4) collected at either ZT10 or ZT22. Amplification of *Gapdh* gene and ROR-deficient liver were used as negative controls. Data represent mean ±SEM, ** P<0.01, *** P<0.001 by ANOVA.
(TIF)

Figure S6 Circadian pattern of expression of *Bmal1*, *Clock*, *Cry1*, *Rev-Erbα*, and *RORγ* was analyzed by QPCR in livers from WT(ND) and *RORγ^−/−^*(ND) mice (n = 3) collected every 4 h over a period of 24 h. Data represent mean ±SD, * P<0.05, ** P<0.01, *** P<0.001 by ANOVA.
(TIF)

Figure S7 RORγ-selective regulation of glucose metabolic genes and inhibition of transactivation in *Gck* and *Gckr* regulatory regions by RORγ-selective antagonist. (A) RORγ activates the binding sites to *Glut2*, *Gys2*, and *Dlat* genes. Huh-7 cells were co-transfected with pGL4 plasmid in which the Luc reporter was under the control of *Glut2* (intron 2), *Gys2* (−256/+59), or *Dlat* (−1151/+22), pCMV-β-Gal, and pCMV10-3xFlag-RORγ expression vector. Luciferase activities were normalized by the one transfected with each reporter plasmid and empty vector. (B) The activation of *Gck* (intron 1) and *Gckr*(−685/+42) regulatory regions by RORγ was inhibited by RORγ-selective antagonist “A” in a dose-responsive manner. Data represent mean ±SEM, * P<0.05 by ANOVA. (C) Circadian expression of *Pepck*, *Glut2*, *Gys2*, *Pklr*, and *Gck* was analyzed by QPCR in liver from WT and *RORα^sg/sg^* mice (n = 4) collected every 4 h over a period of 24 h. (D) Comparison of the expression of RORγ-regulated glucose metabolic genes between livers collected from WT, *RORα^sg/sg^*, *RORγ^−/−^*, and *RORα^sg/sg^RORγ^−/−^*DKO mice at ZT8 or ZT20. *In vivo*, glucose metabolic genes are regulated by RORγ rather than RORα. Data represent mean ±SD, * P<0.05, ** P<0.01, *** P<0.001 by ANOVA.
(TIF)

Table S1 Raw data for ITT, GTT, and PTT experiments.
(XLSX)

Table S2 Gene list of RORγ target genes categorized by GO analysis.
(XLSX)

Table S3 Gene list of RORγ and RORα target genes.
(XLSX)

Table S4 Sequences of primers used in QRT-PCR and ChIP assays.
(DOCX)

## References

[1] Jetten AM (2009) Retinoid-related orphan receptors (RORs): critical roles in development, immunity, circadian rhythm, and cellular metabolism. Nucl Recept Signal 7: e003. 19381306 10.1621/nrs.07003 PMC2670432

[2] Fitzsimmons RL, Lau P, Muscat GE (2012) Retinoid-related orphan receptor alpha and the regulation of lipid homeostasis. J Steroid Biochem Mol Biol 130: 159–168. 21723946 10.1016/j.jsbmb.2011.06.009

[3] Yang XO, Pappu BP, Nurieva R, Akimzhanov A, Kang HS, et al (2008) T helper 17 lineage differentiation is programmed by orphan nuclear receptors ROR alpha and ROR gamma. Immunity 28: 29–39. 18164222 10.1016/j.immuni.2007.11.016 PMC2587175

[4] Huh JR, Leung MW, Huang P, Ryan DA, Krout MR, et al (2011) Digoxin and its derivatives suppress TH17 cell differentiation by antagonizing RORgammat activity. Nature 472: 486–490. 21441909 10.1038/nature09978 PMC3172133

[5] Eberl G, Littman DR (2003) The role of the nuclear hormone receptor RORgammat in the development of lymph nodes and Peyer's patches. Immunol Rev 195: 81–90. 12969312 10.1034/j.1600-065x.2003.00074.x

[6] Solt LA, Kojetin DJ, Burris TP (2011) The REV-ERBs and RORs: molecular links between circadian rhythms and lipid homeostasis. Future Med Chem 3: 623–638. 21526899 10.4155/fmc.11.9 PMC3134326

[7] Akashi M, Takumi T (2005) The orphan nuclear receptor RORalpha regulates circadian transcription of the mammalian core-clock Bmal1. Nat Struct Mol Biol 12: 441–448. 15821743 10.1038/nsmb925

[8] Takeda Y, Kang HS, Angers M, Jetten AM (2011) Retinoic acid-related orphan receptor gamma directly regulates neuronal PAS domain protein 2 transcription in vivo. Nucleic Acids Res 39: 4769–4782. 21317191 10.1093/nar/gkq1335 PMC3113563

[9] Mongrain V, Ruan X, Dardente H, Fortier EE, Cermakian N (2008) Clock-dependent and independent transcriptional control of the two isoforms from the mouse Rorgamma gene. Genes Cells 13: 1197–1210. 19076641 10.1111/j.1365-2443.2008.01237.x

[10] Takeda Y, Jothi R, Birault V, Jetten AM (2012) RORgamma directly regulates the circadian expression of clock genes and downstream targets in vivo. Nucleic Acids Res 40: 8519–8535. 22753030 10.1093/nar/gks630 PMC3458568

[11] Ueda HR, Hayashi S, Chen W, Sano M, Machida M, et al (2005) System-level identification of transcriptional circuits underlying mammalian circadian clocks. Nat Genet 37: 187–192. 15665827 10.1038/ng1504

[12] Liu AC, Tran HG, Zhang EE, Priest AA, Welsh DK, et al (2008) Redundant function of REV-ERBalpha and beta and non-essential role for Bmal1 cycling in transcriptional regulation of intracellular circadian rhythms. PLoS Genet 4: e1000023. 18454201 10.1371/journal.pgen.1000023 PMC2265523

[13] Duez H, Staels B (2010) Nuclear receptors linking circadian rhythms and cardiometabolic control. Arterioscler Thromb Vasc Biol 30: 1529–1534. 20631353 10.1161/ATVBAHA.110.209098 PMC3056213

[14] Cho H, Zhao X, Hatori M, Yu RT, Barish GD, et al (2012) Regulation of circadian behaviour and metabolism by REV-ERB-alpha and REV-ERB-beta. Nature 485: 123–127. 22460952 10.1038/nature11048 PMC3367514

[15] Rey G, Cesbron F, Rougemont J, Reinke H, Brunner M, et al (2011) Genome-wide and phase-specific DNA-binding rhythms of BMAL1 control circadian output functions in mouse liver. PLoS Biol 9: e1000595. 21364973 10.1371/journal.pbio.1000595 PMC3043000

[16] Bass J, Takahashi JS (2010) Circadian integration of metabolism and energetics. Science 330: 1349–1354. 21127246 10.1126/science.1195027 PMC3756146

[17] Eckel-Mahan K, Sassone-Corsi P (2013) Metabolism and the circadian clock converge. Physiol Rev 93: 107–135. 23303907 10.1152/physrev.00016.2012 PMC3781773

[18] Asher G, Schibler U (2011) Crosstalk between components of circadian and metabolic cycles in mammals. Cell Metab 13: 125–137. 21284980 10.1016/j.cmet.2011.01.006

[19] Ripperger JA, Albrecht U (2012) REV-ERB-erating nuclear receptor functions in circadian metabolism and physiology. Cell Res 22: 1319–1321. 22613952 10.1038/cr.2012.81 PMC3434349

[20] Meissburger B, Ukropec J, Roeder E, Beaton N, Geiger M, et al (2011) Adipogenesis and insulin sensitivity in obesity are regulated by retinoid-related orphan receptor gamma. EMBO Mol Med 3: 637–651. 21853531 10.1002/emmm.201100172 PMC3377107

[21] Tinahones FJ, Moreno-Santos I, Vendrell J, Chacon MR, Garrido-Sanchez L, et al (2012) The retinoic acid receptor-related orphan nuclear receptor gamma1 (RORgamma1): a novel player determinant of insulin sensitivity in morbid obesity. Obesity 20: 488–497. 21904299 10.1038/oby.2011.267

[22] Jetten AM, Kang HS, Takeda Y (2013) Retinoic acid-related orphan receptors alpha and gamma: key regulators of lipid/glucose metabolism, inflammation, and insulin sensitivity. Front Endocrinol 4: 1. 10.3389/fendo.2013.00001 PMC3555121 23355833

[23] Rudic RD, McNamara P, Curtis AM, Boston RC, Panda S, et al (2004) BMAL1 and CLOCK, two essential components of the circadian clock, are involved in glucose homeostasis. PLoS Biol 2: e377. 15523558 10.1371/journal.pbio.0020377 PMC524471

[24] Shi SQ, Ansari TS, McGuinness OP, Wasserman DH, Johnson CH (2013) Circadian Disruption Leads to Insulin Resistance and Obesity. Curr Biol 23: 372–381. 23434278 10.1016/j.cub.2013.01.048 PMC3595381

[25] Clark PW, Jenkins AB, Kraegen EW (1990) Pentobarbital reduces basal liver glucose output and its insulin suppression in rats. Am J Physiol 258: E701–707. 2185651 10.1152/ajpendo.1990.258.4.E701

[26] Bass J (2012) Circadian topology of metabolism. Nature 491: 348–356. 23151577 10.1038/nature11704

[27] Burgess SC, Jeffrey FM, Storey C, Milde A, Hausler N, et al (2005) Effect of murine strain on metabolic pathways of glucose production after brief or prolonged fasting. Am J Physiol Endocrinol Metab 289: E53–61. 15797985 10.1152/ajpendo.00601.2004

[28] Bugge A, Feng D, Everett LJ, Briggs ER, Mullican SE, et al (2012) Rev-erbalpha and Rev-erbbeta coordinately protect the circadian clock and normal metabolic function. Genes Dev 26: 657–667. 22474260 10.1101/gad.186858.112 PMC3323877

[29] Kang HS, Angers M, Beak JY, Wu X, Gimble JM, et al (2007) Gene expression profiling reveals a regulatory role for ROR alpha and ROR gamma in phase I and phase II metabolism. Physiol Genomics 31: 281–294. 17666523 10.1152/physiolgenomics.00098.2007

[30] Liu S, Hatano B, Zhao M, Yen CC, Kang K, et al (2011) Role of peroxisome proliferator-activated receptor {delta}/{beta} in hepatic metabolic regulation. J Biol Chem 286: 1237–1247. 21059653 10.1074/jbc.M110.138115 PMC3020731

[31] Chopra AR, Louet JF, Saha P, An J, Demayo F, et al (2008) Absence of the SRC-2 coactivator results in a glycogenopathy resembling Von Gierke's disease. Science 322: 1395–1399. 19039140 10.1126/science.1164847 PMC2668604

[32] Im SS, Kim MY, Kwon SK, Kim TH, Bae JS, et al (2011) Peroxisome proliferator-activated receptor {alpha} is responsible for the up-regulation of hepatic glucose-6-phosphatase gene expression in fasting and db/db Mice. J Biol Chem 286: 1157–1164. 21081500 10.1074/jbc.M110.157875 PMC3020722

[33] Yang X, Downes M, Yu RT, Bookout AL, He W, et al (2006) Nuclear receptor expression links the circadian clock to metabolism. Cell 126: 801–810. 16923398 10.1016/j.cell.2006.06.050

[34] Giguere V, Tini M, Flock G, Ong E, Evans RM, et al (1994) Isoform-specific amino-terminal domains dictate DNA-binding properties of ROR alpha, a novel family of orphan hormone nuclear receptors. Genes Dev 8: 538–553. 7926749 10.1101/gad.8.5.538

[35] Medvedev A, Yan ZH, Hirose T, Giguere V, Jetten AM (1996) Cloning of a cDNA encoding the murine orphan receptor RZR/ROR gamma and characterization of its response element. Gene 181: 199–206. 8973331 10.1016/s0378-1119(96)00504-5

[36] Kurebayashi S, Ueda E, Sakaue M, Patel DD, Medvedev A, et al (2000) Retinoid-related orphan receptor gamma (RORgamma) is essential for lymphoid organogenesis and controls apoptosis during thymopoiesis. Proc Natl Acad Sci USA 97: 10132–10137. 10963675 10.1073/pnas.97.18.10132 PMC27750

[37] Narlikar L, Jothi R (2012) ChIP-Seq data analysis: identification of protein-DNA binding sites with SISSRs peak-finder. Meth Mol Biol 802: 305–322. 10.1007/978-1-61779-400-1_20 PMC4783134 22130889

[38] Jothi R, Cuddapah S, Barski A, Cui K, Zhao K (2008) Genome-wide identification of in vivo protein-DNA binding sites from ChIP-Seq data. Nucleic Acids Res 36: 5221–5231. 18684996 10.1093/nar/gkn488 PMC2532738

