# Supplementary figures and images for: Retinoic Acid-Related Orphan Receptor γ (RORγ): A Novel Participant in the Diurnal Regulation of Hepatic Gluconeogenesis and Insulin Sensitivity
Source: PLoS Genet. 2014 May 15;10(5):e1004331. doi: 10.1371/journal.pgen.1004331 (PMC4022472; doi:10.1371/journal.pgen.1004331)

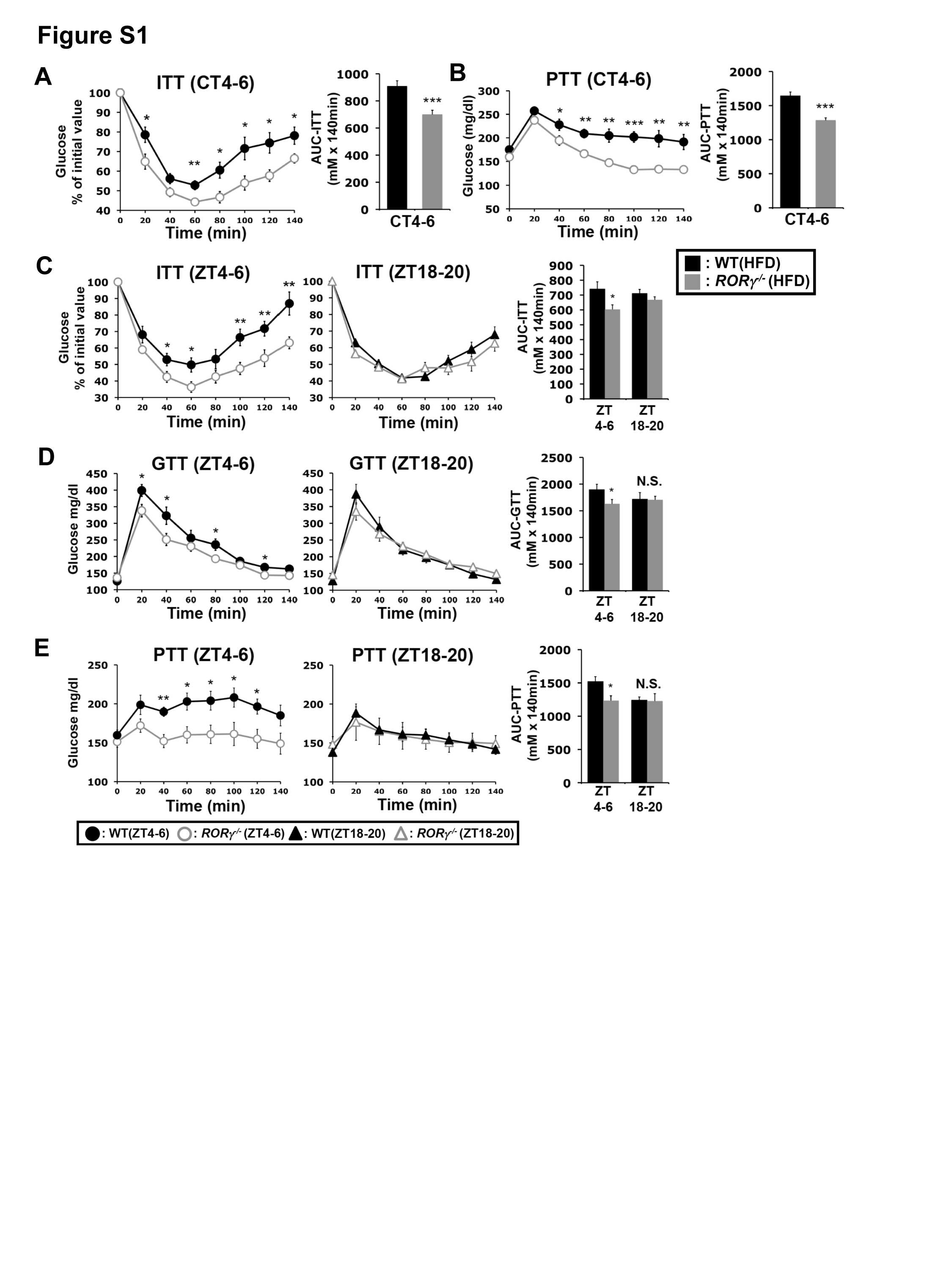

Supplement: Figure S1 — RORγ−/−(HFD) mice exhibited improved insulin sensitivity and hepatic gluconeogenesis under ZT-free condition (constant darkness). ITT (A) and PTT (B) were performed during CT4–6, a subjective daytime, in WT(HFD) and RORγ−/−(HFD) mice (n = 11–12). Mice were kept under constant darkness for 1 day before the start of the experiments. Bar graphs show AUC for ITT and PTT. (C–E) RORγ−/− mice fed a ND exhibited improved insulin sensitivity and glucose tolerance. ITT, GTT, and PTT were performed during ZT4–6 and ZT18–20 in WT(ND) and RORγ−/−(ND) mice (n = 7–9). Bar graphs show AUC for ITT, GTT and PTT. Data represent mean ±SEM, * P<0.05, ** P<0.01, *** P<0.001 by ANOVA. Total AUC for ITT, GTT and PTT in (C–E) was also evaluated by 2-way ANOVA (ITT: Time period: P = 0.1234, Genotype: P = 0.0045; GTT: Time period: P = 0.8575, Genotype: P = 0.0018; PTT: Time period: P = 0.0623, Genotype: P = 0.0472; not shown). (TIF) [file pgen.1004331.s001.tif]

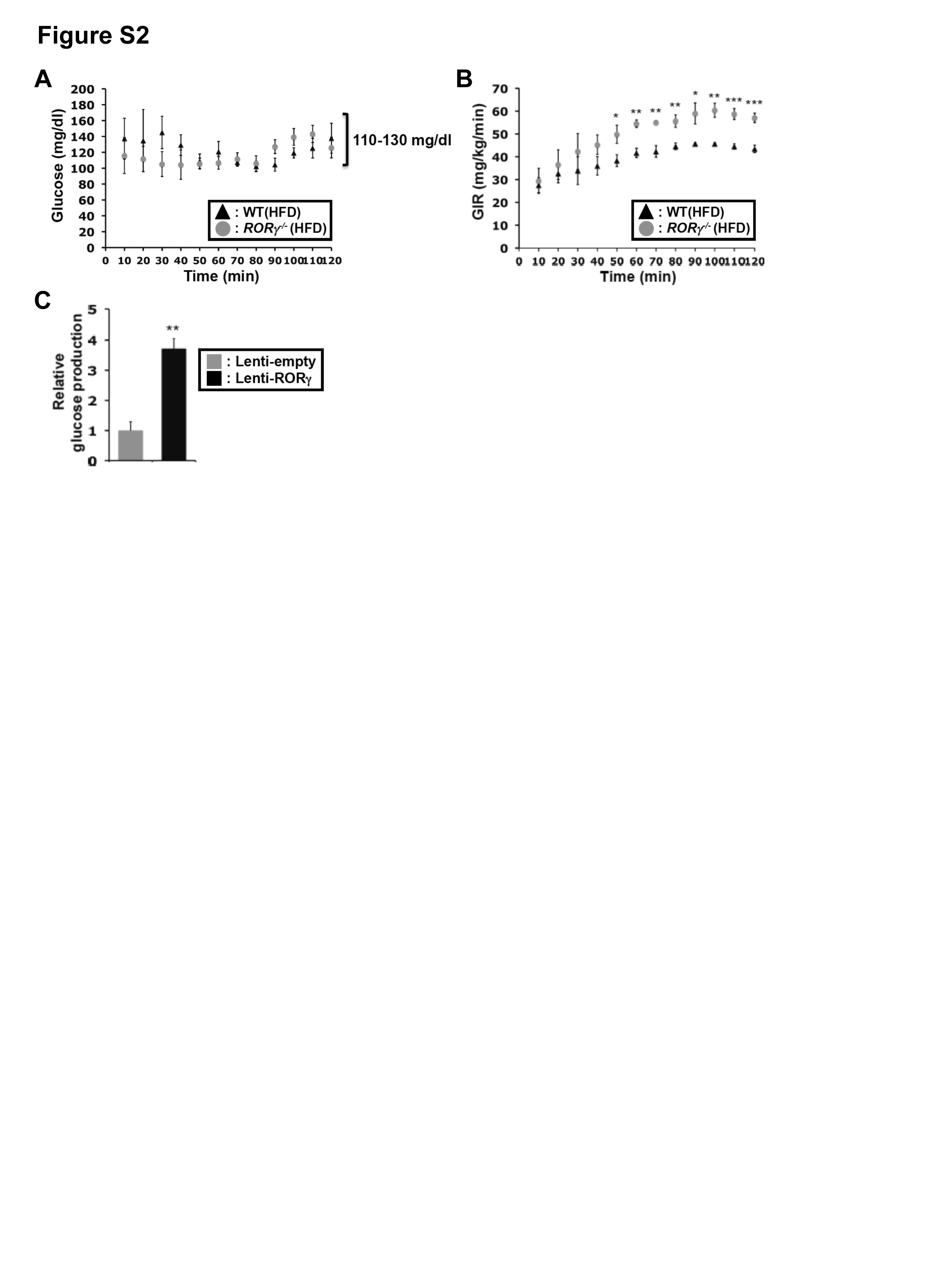

Supplement: Figure S2 — Blood glucose level and GIR during insulin clamp test. (A) Blood glucose levels were measured every 10 min for 2 h during the insulin clamp test. The levels were maintained between 110 to 130 mg/dl. (B) Average GIR during the insulin clamp test. (C) Glucose production in primary RORγ−/− hepatocytes infected with empty or RORγ lentivirus (n = 3). Data represent mean ±SEM, * P<0.05, ** P<0.01, *** P<0.001 by ANOVA. (TIF) [file pgen.1004331.s002.tif]

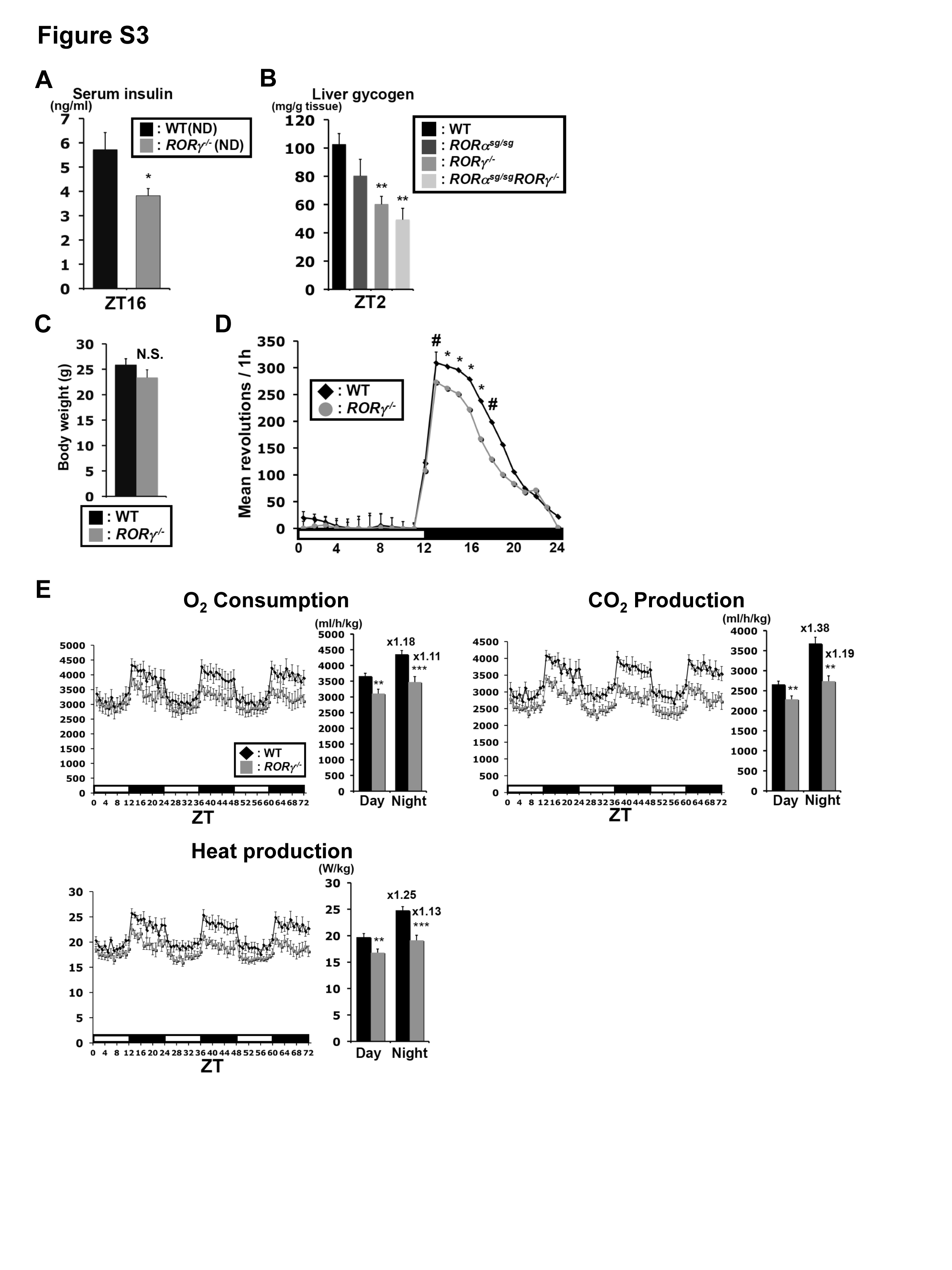

Supplement: Figure S3 — ROR−/− mice exhibit reduced energy expenditure at nighttime. (A) Serum insulin levels were compared between WT(ND) and RORγ−/− (ND) mice (n = 5) at ZT16. (B) Glycogen levels were analyzed in livers from WT(ND), RORαsg/sg(ND), RORγ−/−(ND), and RORαsg/sgRORγ−/−(ND) mice (n = 4) collected at ZT2. Serum insulin and hepatic glycogen levels are reduced in RORγ−/−(ND) mice. Data represent mean ±SEM, * P<0.05, ** P<0.01 by ANOVA. (C) Body weights were not significantly different between WT(ND) and RORγ−/−(ND) mice. (D) Locomoter activity in WT(ND) and RORγ−/−(ND) mice (n = 9–11) was evaluated by the wheel running test. (E) Oxygen consumption (VO2), CO2 production (VCO2), and heat production in WT(ND) (black bars and lines) and RORγ/−(ND) (grey bars and lines) mice (n = 8) were measured during 3 successive days using metabolic cages. The mice were kept under 12 h/12 h light/dark cycles. The numbers indicate fold-increase between day and night in each WT and RORγ/− mice. Data represent mean ±SEM, * P<0.05, ** P<0.01, *** P<0.001 by ANOVA. (TIF) [file pgen.1004331.s003.tif]

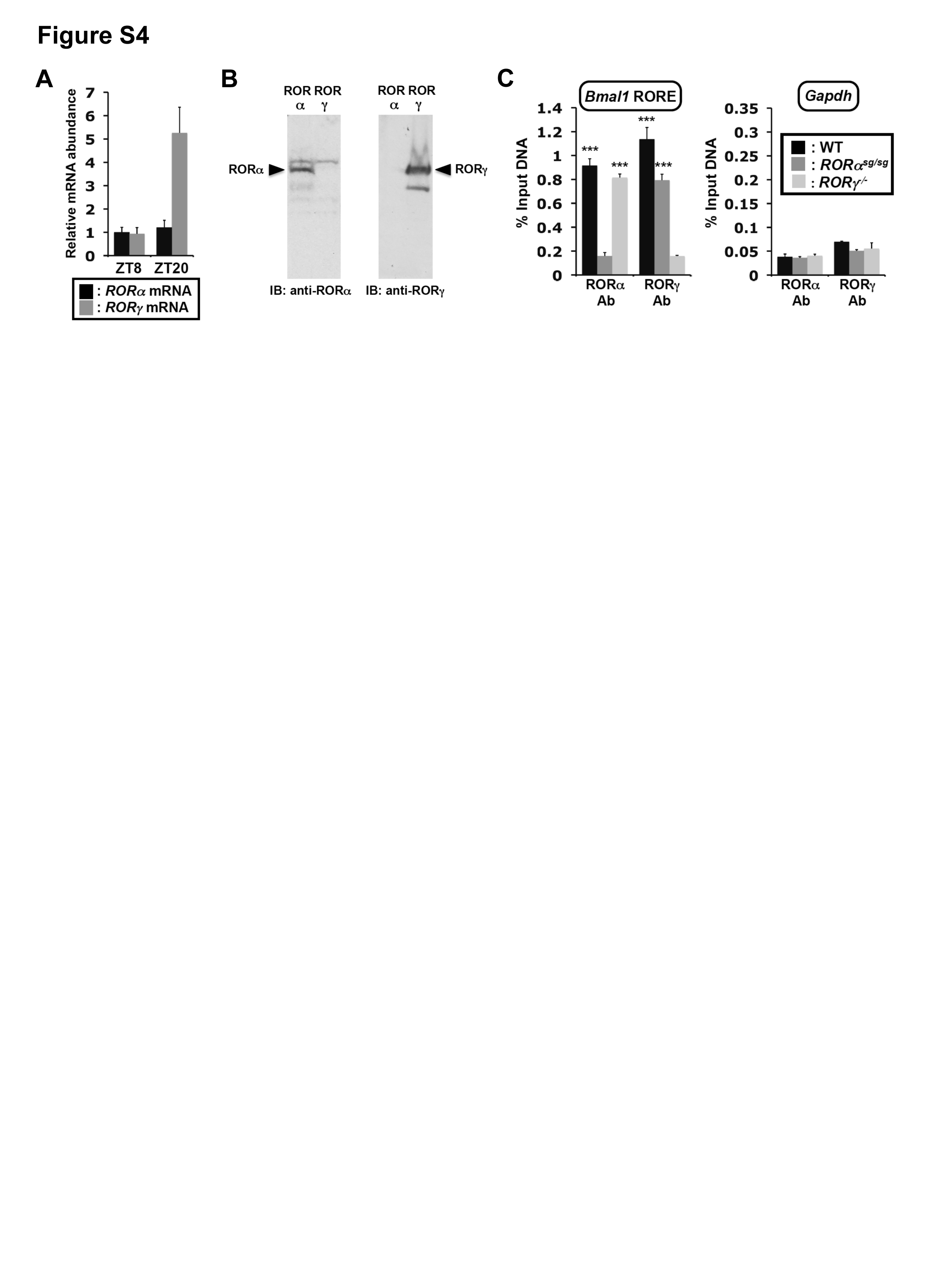

Supplement: Figure S4 — Specificity of anti-ROR antibodies used in ChIP-Seq analysis. (A) RORα and RORγ mRNA expression were compared by QPCR in the liver collected from WT mice at ZT8 and ZT20 (n = 4). (B) Western blot analysis was performed using lysates prepared from HEK293 cells over-expressing RORα or RORγ and antibodies against RORα or RORγ. (C) ChIP-QPCR was performed using each anti-ROR antibody and chromatin prepared from livers of WT, RORαsg/sg, and RORγ−/− mice (n = 4) at ZT22. Amplification of Bmal1 RORE and Gapdh was used as a positive and negative control, respectively. Data represent mean ±SEM, *** P<0.001 by ANOVA. (TIF) [file pgen.1004331.s004.tif]

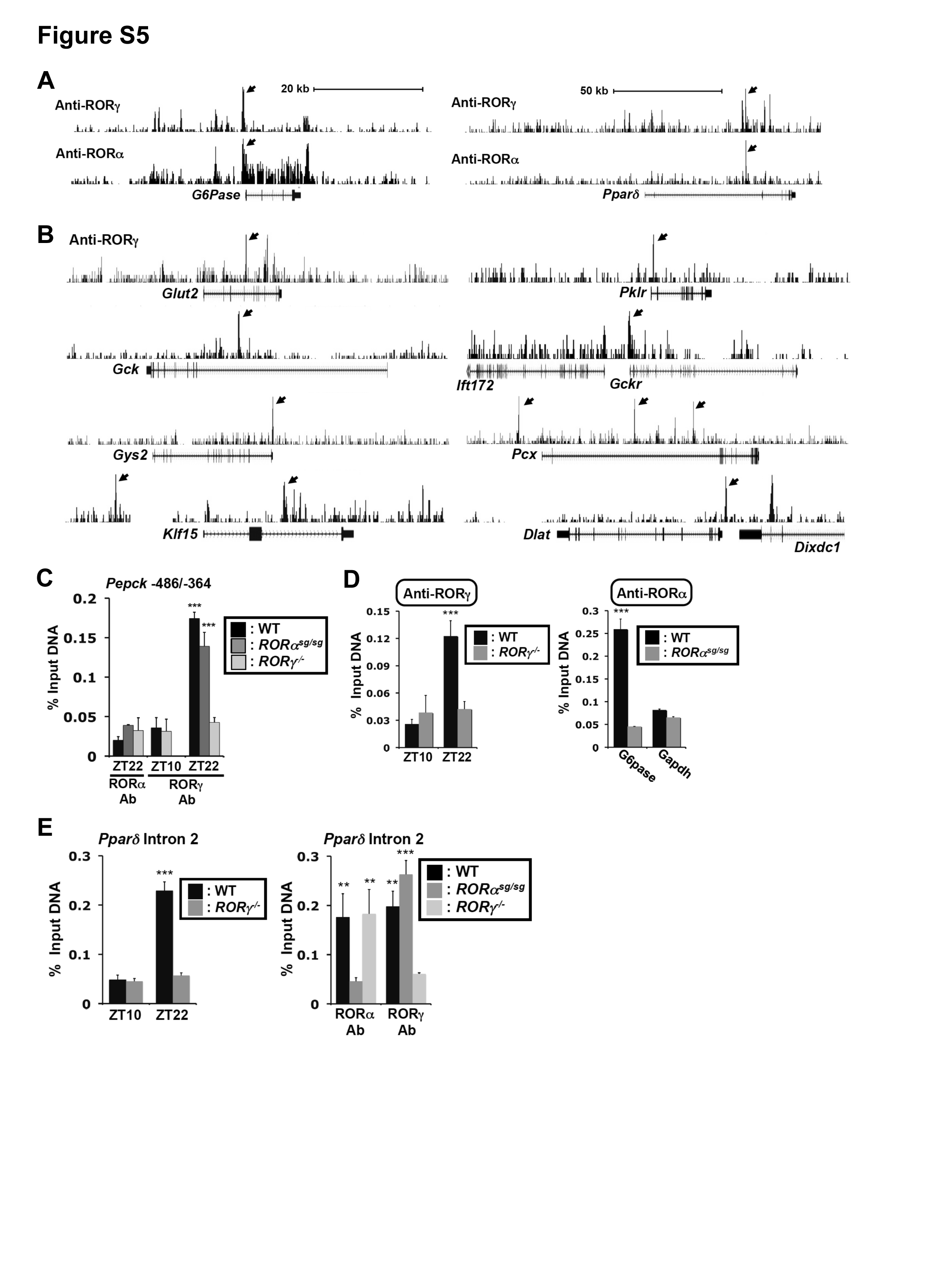

Supplement: Figure S5 — Mapping of RORγ or RORα binding sites to several gene loci in mouse liver. (A, B) UCSC Genome Browser tracks derived from RORγ and RORα ChIP-Seq data are shown in G6pase and Pparδ genes (A), Glut2, Pklr, Gck, Gckr, Gys2, Pcx, Klf15, and Dlat genes (B). (C–E) To confirm ROR binding to Pepck(−486/−364) (C), G6pase(−500/+58) (D), and Pparδ(intron2) (E) ChIP-QPCR was performed using anti-RORγ or -RORα antibody and chromatin prepared from the liver of WT, RORγ−/− or RORαsg/sg mice (n = 4) collected at either ZT10 or ZT22. Amplification of Gapdh gene and ROR-deficient liver were used as negative controls. Data represent mean ±SEM, ** P<0.01, *** P<0.001 by ANOVA. (TIF) [file pgen.1004331.s005.tif]

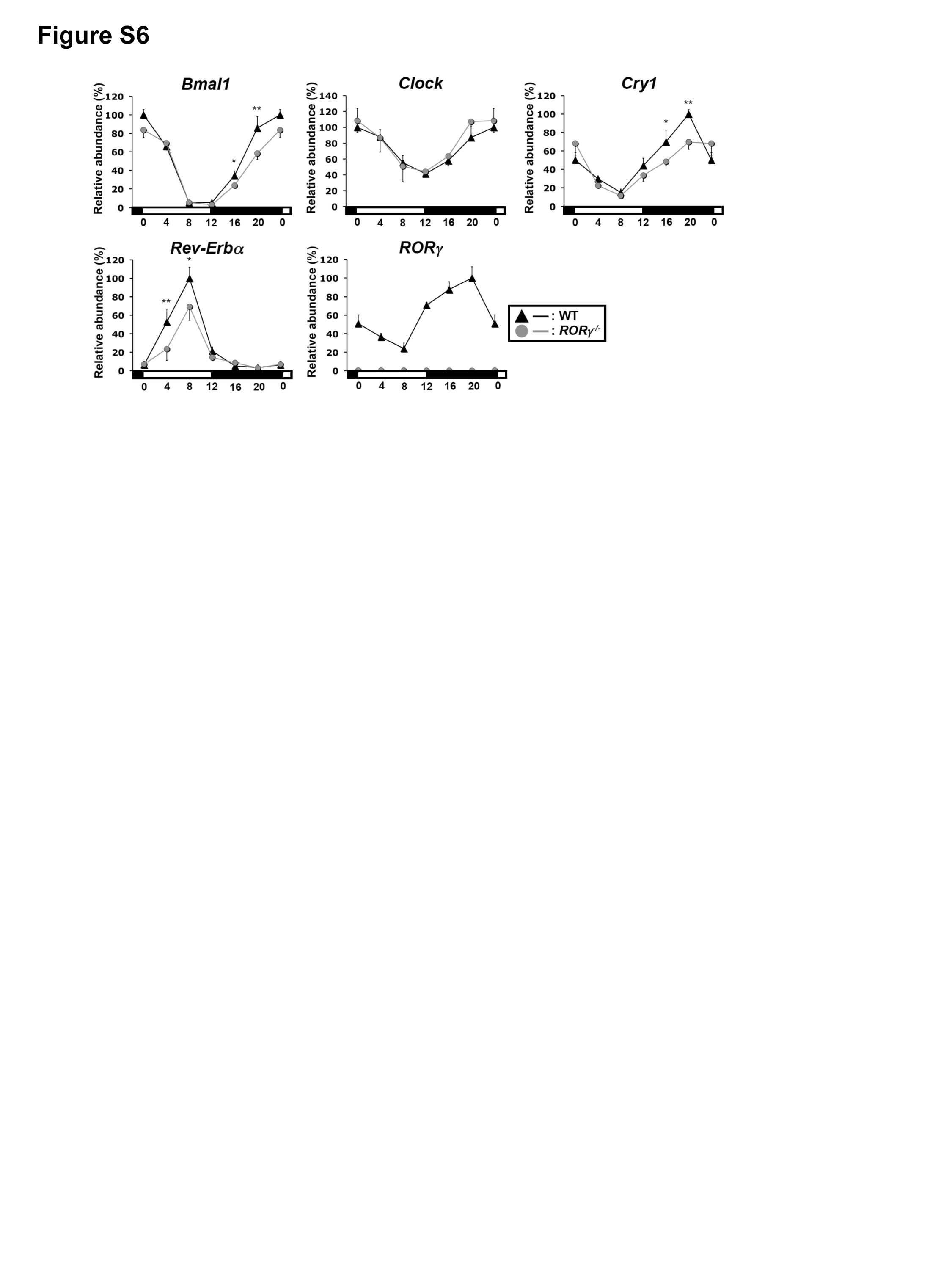

Supplement: Figure S6 — Circadian pattern of expression of Bmal1, Clock, Cry1, Rev-Erbα, and RORγ was analyzed by QPCR in livers from WT(ND) and RORγ−/−(ND) mice (n = 3) collected every 4 h over a period of 24 h. Data represent mean ±SD, * P<0.05, ** P<0.01, *** P<0.001 by ANOVA. (TIF) [file pgen.1004331.s006.tif]

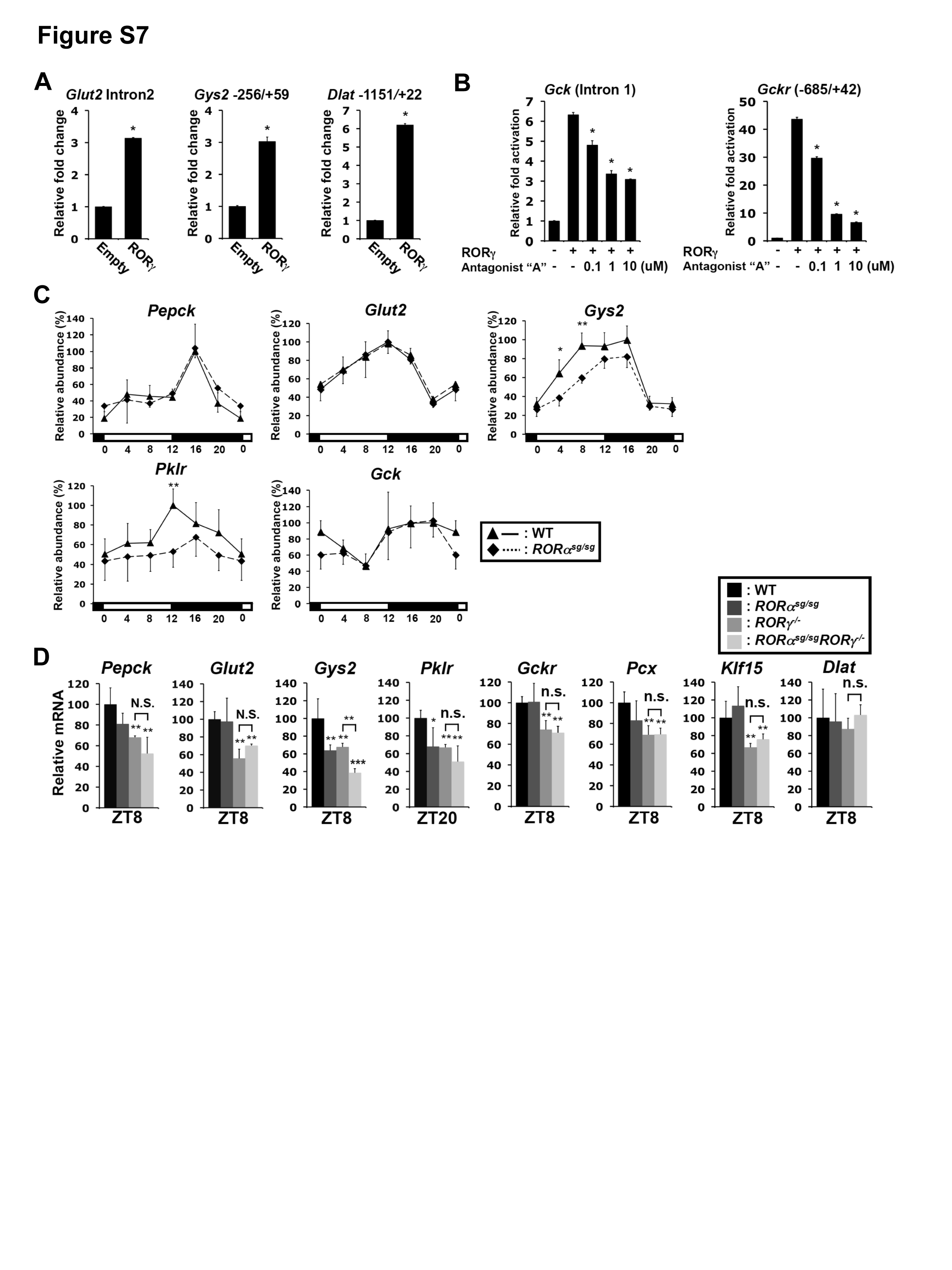

Supplement: Figure S7 — RORγ-selective regulation of glucose metabolic genes and inhibition of transactivation in Gck and Gckr regulatory regions by RORγ-selective antagonist. (A) RORγ activates the binding sites to Glut2, Gys2, and Dlat genes. Huh-7 cells were co-transfected with pGL4 plasmid in which the Luc reporter was under the control of Glut2 (intron 2), Gys2 (−256/+59), or Dlat (−1151/+22), pCMV-β-Gal, and pCMV10-3xFlag-RORγ expression vector. Luciferase activities were normalized by the one transfected with each reporter plasmid and empty vector. (B) The activation of Gck (intron 1) and Gckr(−685/+42) regulatory regions by RORγ was inhibited by RORγ-selective antagonist “A” in a dose-responsive manner. Data represent mean ±SEM, * P<0.05 by ANOVA. (C) Circadian expression of Pepck, Glut2, Gys2, Pklr, and Gck was analyzed by QPCR in liver from WT and RORαsg/sg mice (n = 4) collected every 4 h over a period of 24 h. (D) Comparison of the expression of RORγ-regulated glucose metabolic genes between livers collected from WT, RORαsg/sg, RORγ−/−, and RORαsg/sgRORγ−/−DKO mice at ZT8 or ZT20. In vivo, glucose metabolic genes are regulated by RORγ rather than RORα. Data represent mean ±SD, * P<0.05, ** P<0.01, *** P<0.001 by ANOVA. (TIF) [file pgen.1004331.s007.tif]
