# Supplementary material for: Retinoic Acid-Related Orphan Receptor γ (RORγ): A Novel Participant in the Diurnal Regulation of Hepatic Gluconeogenesis and Insulin Sensitivity
Source: PLoS Genet. 2014 May 15;10(5):e1004331. doi: 10.1371/journal.pgen.1004331 (PMC4022472; doi:10.1371/journal.pgen.1004331)
Supplement: Table S4 — Sequences of primers used in QRT-PCR and ChIP assays. (DOCX) [file pgen.1004331.s011.docx]

**Table S4.** Sequences of primers used in QRT-PCR and ChIP assays

| **Gene** | **Sense primer** | **Antisense pimer** |
| --- | --- | --- |
| *mRORγ* | ACTACGGGGTTATCACCTGTGAG | GTGCAGGAGTAGGCCACATTAC |
| *mRORα* | GCACCTGACCGAAGACGAAA | GAGCGATCCGCTGACATCA |
| *mGapdh* | AGTATGACTCCACTCACGGCAAAT | GTCTCGCTCCTGGAAGATGGT |
| *mG6pase* | GGACACCGACTACTACAGCAACAG | GCATGGCCAGAGGGACTTC |
| *mPepck* | CGATGACATTGCCTGGATGAAG | TCTTCACTGAGGTGCCAGGAG |
| *mGlut2* | GTGTCTGCTACTGCTCTTCTGTC | GACATCCTCAGTTCCTCTTAGTCTC |
| *mPklr* | TCTTGGCTCAGAAGATGATGATTG | TGGTCGAGCCTTAGTGATCATG |
| *mGck* | CTTCACCTTCTCCTTCCCTG | ATCTCAAAGTCCCCTCTCCT |
| *mGckr* | GAGAGTGGTCGTTATAGGCATTTC | GTTATCCATGCAGTAGTCCATCTG |
| *mGys2* | TGGGTCTTTAACTGCCTGGTTC | TGTTTACCGTCTGCGTGGTC |
| *mPparδ* | AGAACCGCAACAAGTGTCAGTAC | CATCCGTCCAAAGCGGATAG |
| *mDlat* | ACATTGACTCTTTTGTGCCTTCTAAG | ATGTCTGTGAAGACACCTGCAG |
| *mPcx* | CAGTCCTGTGGATCCTGCTG | AGAAGGATGTCCCTGAAACCAG |
| *mKlf15* | CACACAAAATGTGCAGCCAGCAAC | GCCATAGTGAGTCCTTACACAACATTTG |
| *mG6paseRORE* | GCAGCCTCTAGCACTGTCAAG | AACAGCCTGATCGCCATTG |
| *mPepck*  *RORE* | AGAAGTCTCATGGCTCAGAGCTG | GTTGCTGGCTGCACATTTTGTGTG |
| *mPparδ*  *RORE* | CACACAAAATGTGCAGCCAGCAAC | GCCATAGTGAGTCCTTACACAACATTTG |
